# Efficacy and safety of Zolbetuximab plus chemotherapy for advanced CLDN18.2-positive gastric or gastro-oesophageal adenocarcinoma: a meta-analysis of randomized clinical trials

**DOI:** 10.1186/s12885-024-11980-w

**Published:** 2024-02-21

**Authors:** Francisco Cezar Aquino de Moraes, Eric Pasqualotto, Matheus Pedrotti Chavez, Rafael Oliva Morgado Ferreira, Tiago Biachi De Castria, Rommel Mario Rodríguez Burbano

**Affiliations:** 1 Federal University of Pará, 66073-005 Belém, Pará Brazil; 2 Federal University of Santa Catarina, 88040-900 Florianópolis, Santa Catarina Brazil; 3 Moffitt Cancer Center, 12902 USF Magnolia Drive, 33612 Tampa, FL USA; 4 Morsani College of Medicine, University of South Florida, 12901 Bruce B. Downs Blvd., 33612 Tampa, FL USA; 5 Ophir Loyola Hospital, 66063-240 Belém, Pará Brazil

**Keywords:** Claudin-18 isoform 2, Zolbetuximab, Gastric adenocarcinoma, Adenocarcinoma of the gastro-oesophageal junction, Oesophageal adenocarcinoma

## Abstract

**Background:**

The benefit of adding Zolbetuximab to the treatment in patients with Claudin-18 isoform 2 (CLDN18.2)-positive, human epidermal growth factor receptor 2-negative, locally advanced unresectable or metastatic gastric or gastro-oesophageal junction adenocarcinoma (GC/GEJ) is not yet fully elucidated.

**Methods:**

We searched PubMed, Embase and Cochrane databases for randomized controlled trials (RCTs) that investigated Zolbetuximab plus chemotherapy versus chemotherapy alone for GC or GEJ adenocarcinoma. We computed hazard-ratios (HRs) or odds-ratios (ORs) for binary endpoints, with 95% confidence intervals (CIs).

**Results:**

Three studies and 1,233 patients were included. Comparing with Zolbetuximab plus chemotherapy versus chemotherapy alone, progression-free survival (PFS) rate (HR 0.64; 95% CI 0.49–0.84; *p* < 0.01) and overall survival (OS) rate (HR 0.72; 95% CI 0.62–0.83; *p* < 0.01) were significant in favor of the Zolbetuximab group. Regarding effectiveness, the Objective Response Rate (ORR) was (OR 1.15; 95% CI 0.87–1.53; *p* = 0.34).

**Conclusions:**

In this comprehensive systematic review and meta-analysis of RCTs, the incorporation of Zolbetuximab alongside chemotherapy offers a promising prospect for reshaping the established treatment paradigms for patients diagnosed with advanced CLDN18.2-positive GC/GEJ cancer.

**Supplementary Information:**

The online version contains supplementary material available at 10.1186/s12885-024-11980-w.

## Introduction

Gastric adenocarcinoma (GC) is the fifth most diagnosed type of cancer in the world and, together with adenocarcinomas of the gastro-oesophageal junction (GEJ) and oesophageal adenocarcinomas (EC), has a high mortality rate [1, 2]. Because of their aggressiveness and non-specific symptoms, these tumors are usually diagnosed at a late stage and the prognosis is poor, representing some of the greatest unmet medical needs [2–4]. The standard first-line therapy for human epidermal growth factor receptor 2-negative (HER2-), advanced, unresectable or metastatic GC/GEJ/EC is platinum-fluoropyrimidine-based chemotherapy [5–9]. Platinum-fluoropyrimidine-based chemotherapy resulting in a median overall survival (OS) duration of about 12 months [2, 10–14].

The addition of targeted therapies to chemotherapy improves survival for GC/GEJ, but there are currently few validated molecular targets for this scenario. Treatment with programmed death-ligand 1 (PD-L1) such as nivolumab is approved first-line in combination with chemotherapy in more than 50 countries, but its efficacy is linked to a combined positive score (CPS) of 5 or more [15–17]. The addition of pembrolizumab (PD-L1) to trastuzumab and first-line chemotherapy in the phase III KEYNOTE-811 [18] clinical trial showed an increase in objective response rate (ORR) of 74.4% versus 51.9% for trastuzumab and chemotherapy alone for GC. Currently an unmet need is the development of targeted therapies for patients with HER2-negative advanced, unresectable or metastatic GC/GEJ/EC.

Claudin-18 isoform 2 (CLDN18.2) is a tight junction protein that regulates the polarity and permeability of epithelial layers, which is frequently expressed in normal gastric mucosa cells and is maintained in the majority of GC/GEJ/EC adenocarcinomas [19–22]. During malignant transformation, cell polarity is lost, causing CLDN18.2 to become exposed on the cell surface and thus remain available for monoclonal antibody binding, making it a promising emerging therapeutic target [19, 23–25].

Zolbetuximab (IMAB363) is a first-in-class chimeric monoclonal immunoglobulin G1 antibody that targets CLDN18.2 and mediates tumour cell death by antibody-dependent cytotoxicity (ADCC) and complement-dependent cytotoxicity (CDC) in GC/GEJ/EC CLDN18.2-positive adenocarcinoma cells [23, 26]. Pre-clinical studies have shown significant therapeutic synergism when associated with cytotoxic agents [26]. The phase IIa MONO study showed anti-tumour action and good tolerability when Zolbetuximab was administered as a single agent in heavily pre-treated advanced GC/GEJ CLDN18.2-positive patients [24].

Therefore, in this systematic review and meta-analysis of randomized clinical trials (RCTs), we aim to clarify the benefits for progression-free survival (PFS), OS, response and safety using Zolbetuximab plus chemotherapy verus chemotherapy alone in advanced CLDN18.2-positive GC/GEJ/EC.

## Methods

### Protocol and registration

This systematic review followed the Preferred Reporting Items for Systematic Reviews and Meta-Analysis (PRISMA) guidelines [27]. The protocol was registered in the International Prospective Register of Systematic Reviews (PROSPERO) with registration number CRD42023455827.

### Eligibility criteria

Studies that met the following eligibility criteria were included: (1) RCTs; (2) comparison of Zolbetuximab plus chemotherapy versus chemoterapy alone; (3) adult patients with histologically confirmed adenocarcinoma of the stomach, esophagus or GEJ; (4) locally advanced unresectable or metastatic disease; (5) CLDN18.2 expression confirmed by immunohistochemistry; (6) HER2 negative, HER2 unknown or HER2 positive status but not eligible to trastuzumab therapy; (7) Eastern Cooperative Oncology Group (ECOG) performance-status score of 0–1 (on a 5-point scale in which higher scores reflect greater disability; and (8) life expectancy ≥ 12 weeks. We excluded studies (1) with overlapping populations; (2) without any outcomes of interest; and (3) with unpublished complete results. Inclusion and exclusion criteria for the RCTs included in this systematic review and meta-analysis are detailed in Supplementary Table 1.

Thus, we sought to answer the following question: How effective is the addition of Zolbetuximab to chemotherapy versus chemotherapy alone for treatment of CLDN18.2-positive, HER2-negative, locally advanced unresectable or metastatic GC or GEJ adenocarcinoma?

### Search strategy and data extraction

Pubmed, Cochrane Library and Embase were systematically searched on August 20, 2023. The search strategy is detailed in Supplementary Table 1. In addition, backwards snowballing was performed aiming the inclusion of additional studies. Duplicate articles were removed, using both automated and manual methods. Subsequently, two reviewers (E.P. and M.P.C.) independently analyzed the titles and abstracts of the identified articles. Disagreements were resolved by consensus between the two authors and senior author (E.P., M.P.C. and R.M.R.B).

The following baseline characteristics were extracted: (1) ClinicalTrials.gov Identifier and study design; (2) number of patients allocated for each arm; (3) regimen details in experimental and control arm; and (4) main patient’s characteristics; (1) PFS, defined as the time from patient randomization to disease progression or death from any cause; (2) OS, defined as the period of time, from the randomization, that patients are still alive; (3) Radiographic response, according to the Response Evaluation Criteria for Solid Tumors (RECIST), version 1.1 [28] and (4) adverse events, defined as an unwanted effect of a treatment, which were evaluated by the Common Terminology Criteria for Adverse Events, version 5.0, in the included RCTs [29]. Two authors (E.P. and R.O.M.F.) collected pre-specified baseline characteristics and outcome data.

### Endpoints

Outcomes of interest were: (1) PFS; (2) OS; (3) complete response (CR); (4) partial response (PR); (5) stable disease (SD); (6) progressive disease (PD); (7) objective response rate (ORR); (8) disease control rate (DCR); (9) any grade or grade ≥ 3 of all treatment-emergent events; patients with any grade or grade ≥ 3 of (10) nausea; (11) vomiting; (12) decreased appetite; (13) diarrhea; (14) neutropenia; (15) anaemia; (16) fatigue; (17) asthenia; (18) abdominal pain; (9 weight decreased; (20) pyrexia; (21) oedema peripheral; (22) aspartate aminotransferase increased; (23) alanine aminotransferase increased; and (24) thrombocytopenia.

### Risk of bias assessment

The quality assessment of individual RCTs was carried out using the Cochrane Collaboration tool for assessing risk of bias in randomized trials (RoB 2) [30]. Two authors (E.P. and R.M.O.F.) independently conducted the assessment, and disagreements were resolved by consensus. For each trial, a risk of bias score was assigned, indicating whether it was at a high, low, or unclear risk of bias across five domains: randomization process, deviations from intended interventions, missing outcomes, measurement of outcomes, and selection of reported results [31].

### Assessment of publication bias

Potential publication bias was evaluated through visual inspection of funnel plots and analysis of the control lines. No quantitative assessment of small studies or publication bias was performed due to the small number of included studies.

### Sensitivity analyses

Leave-one-out procedures were used to identify influential studies and their effect on the pooled estimates, evaluating the heterogeneity. This procedure was carried out removing data from one study and reanalyzing the remaining data, confirming that the pooled effect-sizes did not result from a single-study dominance.

### Statistical analysis

Binary endpoints were evaluated with Hazard-ratios (HRs) or odds-ratios (ORs), with 95% confidence intervals (CIs). The Cochrane *Q*-test and I^2^ statistics were used to assess heterogeneity; P values > 0.10 and I^2^ values > 25% were considered to indicate significance for heterogeneity [32]. The Sidik-Jonkman estimator was used to calculate the tau^2^ variance between studies [33]. We used DerSimonian and Laird random-effect models for all endpoints [34]. Statistical analyses were performed using R statistical software, version 4.2.3 (R Foundation for Statistical Computing).

## Results

### Study selection and baseline characteristics

**Fig. 1:**
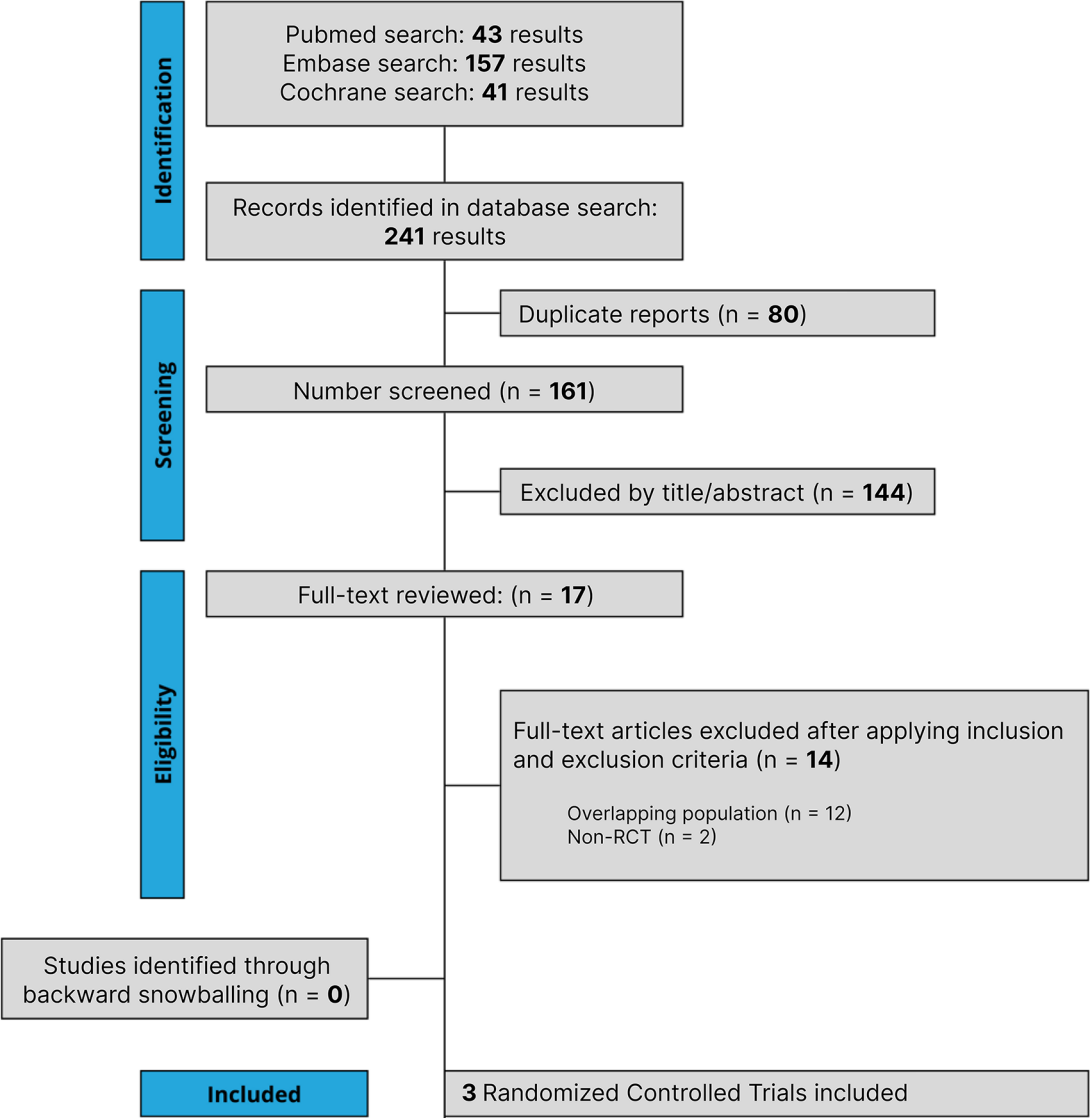
PRISMA flow diagram of study screening and selection

The initial search yielded 241 results, as detailed in Fig. 1. After the removal of duplicate records, and the assessment of the studies based on title and abstract, 17 full-text remained for full review according to prespecified criteria. Of these, three RCTs were included [35–37], comprising 1,233 patients. A total of 614 patients with advanced CLDN18.2-positive GC/GEJ/EC were randomized to Zolbetuximab plus chemotherapy, while 619 received chemotherapy alone. The median age ranged from 57.0 to 62.0 years. 769 (62.4%) patients were men and 464 (37.6%) were female. 459 patients had diffuse classification and 255 had Lauren’s intestinal classification. The primary site of 987 patients was the stomach. 504 patients had an ECOG performance-status score of 0 and 716 patients had a score of 1. Study and participant characteristics are detailed in Table 1 and Supplementary Table S3.

**Table 1:** Design and characterístics of studies included in the meta-analysis

Study	Design/NCT	Intervention treatment	Follow-up (median)	Number of participants IG/CG, n	Median age (IQR or range) IG/CG, years	Male IG/CG, no. (%)	Primary site, n (%)		Organs with metastases, n (%)		Lauren classification, n (%)	
							IG	CG	IG	CG	IG	CG
FAST 2021 [35]	Phase II/ NCT01630083	Zolbetuximab plus EOX	54.7 months	77/84	59 (range 22–77)/ 57 (range 24–73)	47 (61.0)/ 56 (66.7)	Stomach: 62 (80.5) GEJ: 13 (16.9) Oesophagus: 2 (2.6)	Stomach: 68 (81.0) GEJ: 12 (14.3) Oesophagus: 4 (4.8)	NA	NA	Diffuse 35 (45.5) Intestinal 26 (33.8) Mixed 10 (13.0) Unknown 6 (7.8)	Diffuse 38 (45.2) Intestinal 27 (32.1) Mixed 11 (13.1) Unknown 8 (9.5)
GLOW 2023 [36]	Phase III/ NCT03653507	Zolbetuximab plus CAPOX	12.62 months	254/253	61.0 (range 22–82)/ 59.0 (range 21–83)	159 (62.6)/ 156 (61.7)	Stomach: 219 (86.2) GEJ: 35 (13.8)	Stomach: 209 (82.6) GEJ: 44 (17.4)	0–2: 189 (74.4) ≥ 3: 65 (25.6)	0–2: 188 (74.3) ≥ 3: 65 (25.7)	Diffuse 87 (34.4) Intestinal 36 (14.2) Mixed 20 (7.9) Unknown 76 (30.0) Other 34 (13.4)§	Diffuse 100 (39.5) Intestinal 41 (16.2) Mixed 21 (8.3) Unknown 64 (25.3) Other 27 (10.7)§
SPOTLIGHT 2023 [37]	Phase III/ NCT03504397	Zolbetuximab plus mFOLFOX6	NA	283/282	62·0 (IQR 51·0–69·0)/ 60·0 (IQR 50·0–69·0)	176 (62)/ 175 (62)	Stomach: 219 (77) GEJ: 64 (23)	Stomach: 210 (74) GEJ: 72 (26)	0–2: 219 (77) ≥ 3: 64 (23)	0–2: 219 (78) ≥ 3: 63 (22)	Diffuse 82 (29) Intestinal 70 (25) Mixed 31 (11) Unknown 49 (17) Other 50 (18)☨	Diffuse 117 (41) Intestinal 66 (23) Mixed 13 (5) Unknown 40 (14) Other 42 (15)☨

§ Missing Lauren classification for 1 patient in Zolbetuximab plus CAPOX group. ☨ Missing Lauren classification for 1 patient in Zolbetuximab plus mFOLFOX6 group and 4 patients in placebo plus mFOLFOX6 group. CAPOX, Capecitabine and Oxaliplatin; CG, control group; EOX, Epirubicin, Oxaliplatin and Capecitabine; IG, intervention group; IQR, interquartile range; mFOLFOX6, 5-fluorouracil, folinic acid and oxaliplatin; NA, not available; NCT, National Clinical Trial

### Progression-free survival

**Fig. 2:**
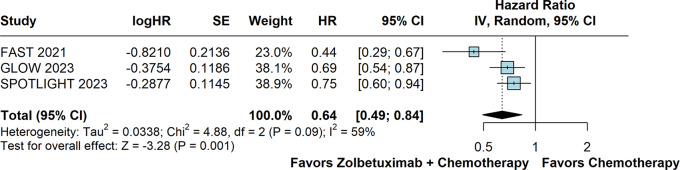
Progression-free survival of patients with advanced CLDN18.2-positive gastric or gastro-oesophageal adenocarcinoma treated with Zolbetuximab plus chemotherapy versus chemotherapy alone

PFS was significantly prolonged in patients randomized to receive Zolbetuximab plus chemotherapy versus chemotherapy alone (HR 0.64; 95% CI 0.49–0.84; *p* < 0.01; I²=59%; Fig. 2).

### Overall survival

**Fig. 3:**
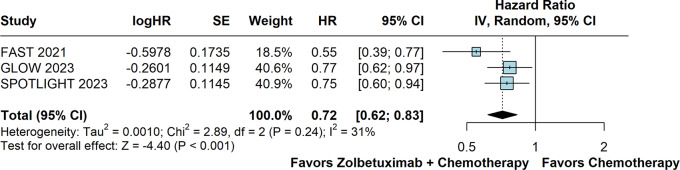
Overall survival of patients with advanced CLDN18.2-positive gastric or gastro-oesophageal adenocarcinoma treated with Zolbetuximab plus chemotherapy versus chemotherapy alone

OS was significantly prolonged in patients randomized to receive Zolbetuximab plus chemotherapy versus chemotherapy alone (HR 0.72; 95% CI 0.62–0.83; *p* < 0.01; I²=31%; Fig. 3).

### Radiographic response

**Fig. 4:**
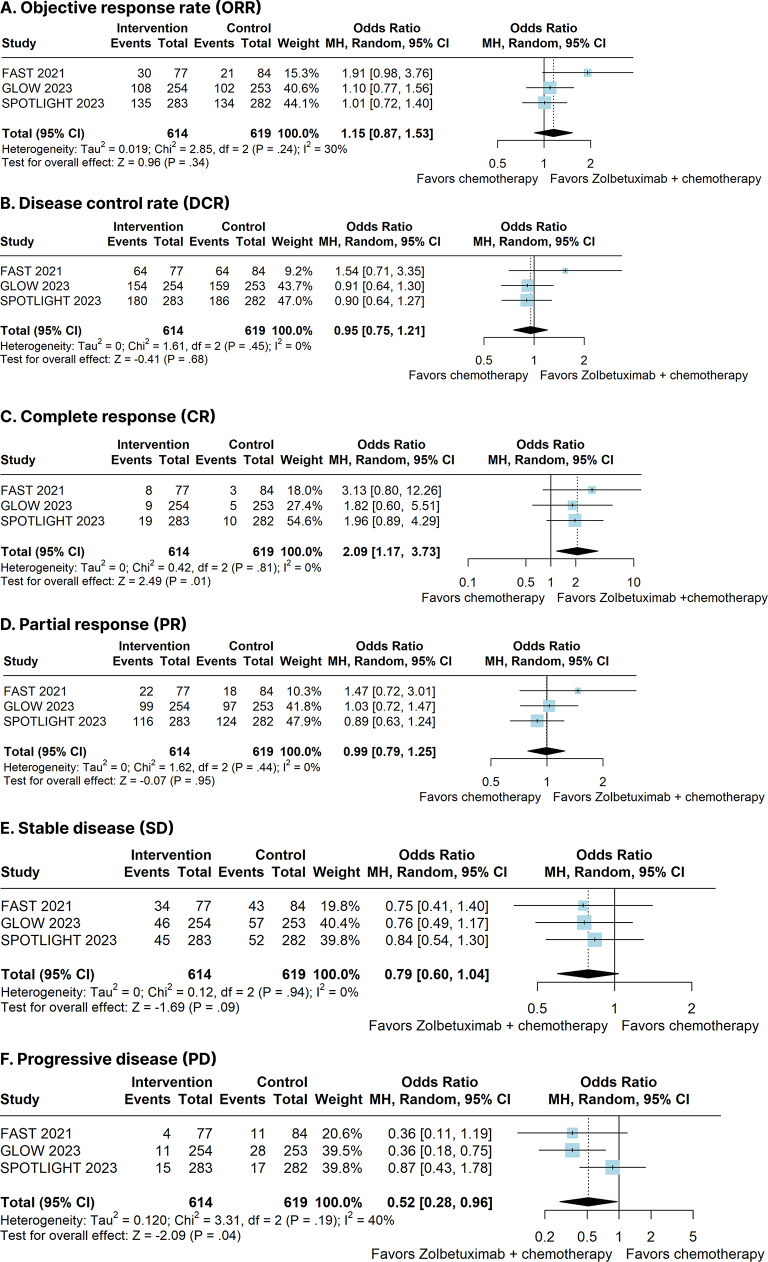
Radiographic response of patients with advanced CLDN18.2-positive gastric or gastro-oesophageal adenocarcinoma treated with Zolbetuximab plus chemotherapy versus chemotherapy alone. **(A)** Objective response rate (ORR). **(B)** Disease control rate (DCR). **(C)** Complete response (CR). **(D)** Partial response (PR). **(E)** Stable disease (SD). **(F)** Progressive disease (PD)

Zolbetuximab plus chemotherapy significantly increased CR (OR 2.09; 95% CI 1.17–3.73; *p* = 0.01; I²=0%; Fig. 4C) and reduced PD (OR 0.52; 95% CI 0.28–0.96; *p* = 0.04; I²=40%; Fig. 4F). There was no significant different between groups for ORR (OR 1.15; 95% CI 0.87–1.53; *p* = 0.34; I²=30%; Fig. 4A), PR (OR 0.99; 95% CI 0.79–1.25; *p* = 0.95; I²=0%; Fig. 4D), SD (OR 0.79; 95% CI 0.60–1.04; *p* = 0.09; I²=0%; Fig. 4E), and DCR (OR 0.95; 95% CI 0.75–1.21; *p* = 0.68; I²=0%; Fig. 4B).

### Safety

**Fig. 5:**
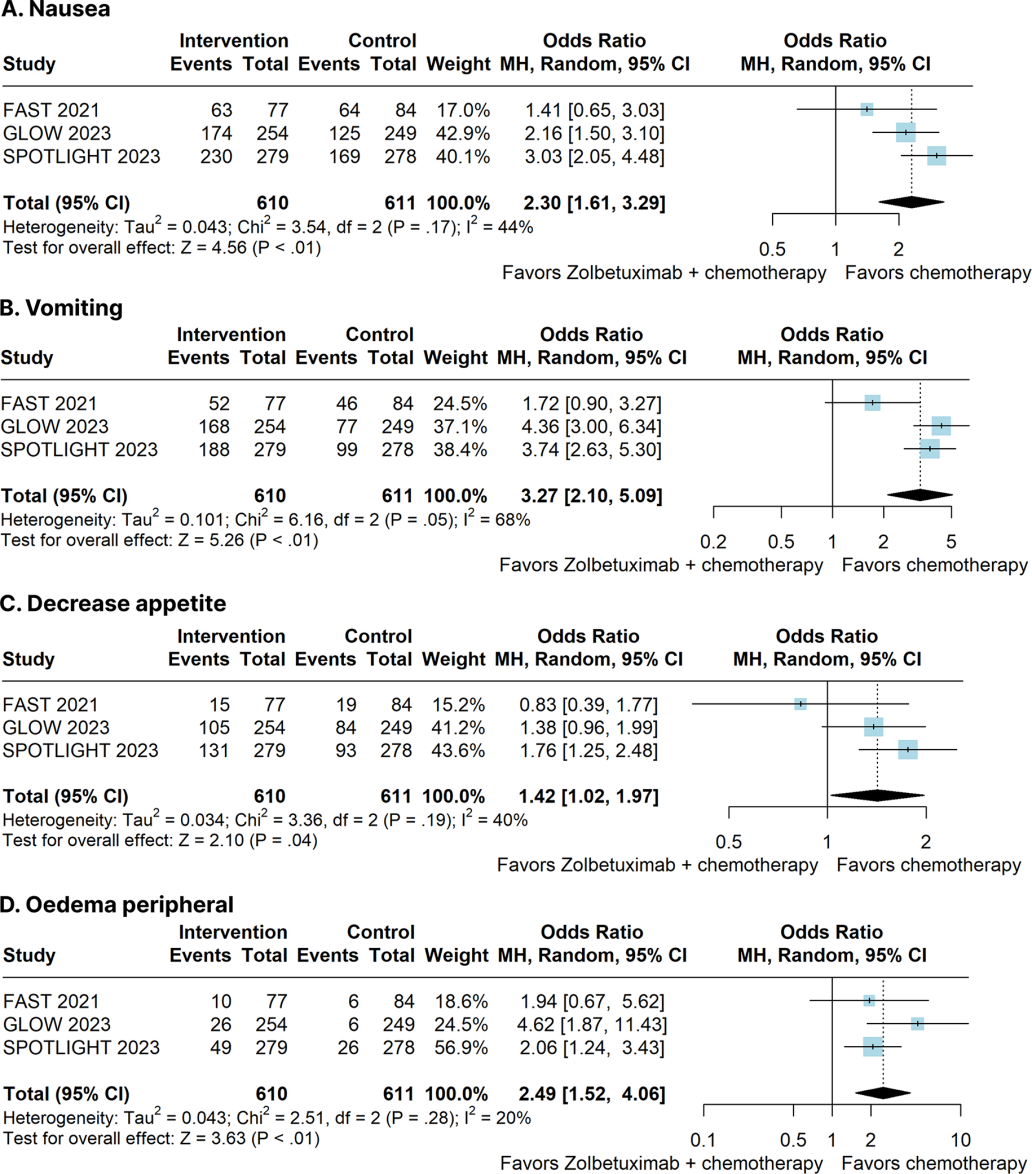
Any grade of adverse events of patients with advanced CLDN18.2-positive gastric or gastro-oesophageal adenocarcinoma treated with Zolbetuximab plus chemotherapy versus chemotherapy alone. **(A)** Nausea. **(B)** Vomiting. **(C)** Decrease appetite. **(D)** Oedema peripheral

Zolbetuximab plus chemotherapy significantly increased any grade of nausea (OR 2.30; 95% CI 1.61–3.29; *p* < 0.01; I²=44%; Fig. 5A), vomiting (OR 3.27; 95% CI 2.10–5.09; *p* < 0.01; I²=68%; Fig. 5B), decrease appetite (OR 1.42; 95% CI 1.02–1.97; *p* = 0.04; I²=40%; Fig. 5C), and oedema peripheral (OR 2.49; 95% CI 1.52–4.06; *p* < 0.01; I²=20%; Fig. 5D). There was no significant difference between groups for any grade of all treatment-emergent events (OR 1.05; 95% CI 0.17–6.70; *p* = 0.96; I²=50%; Supplementary Figure S1), diarrhea (OR 0.73; 95% CI 0.51–1.06; *p* = 0.10; I²=53%; Supplementary Figure S2), neutropenia (OR 1.28; 95% CI 0.99–1.66; *p* = 0.06; I²=0%; Supplementary Figure S3), anaemia (OR 1.00; 95% CI 0.80–1.27; *p* = 0.97; I²=0%; Supplementary Figure S4), fatigue (OR 0.94; 95% CI 0.61–1.45; *p* = 0.78; I²=54%; Supplementary Figure S5), asthenia (OR 1.13; 95% CI 0.85–1.51; *p* = 0.39; I²=0%; Supplementary Figure S6), abdominal pain (OR 0.81; 95% CI 0.56–1.18; *p* = 0.28; I²=37%; Supplementary Figure S7), weight decrease (OR 1.34; 95% CI 0.81–2.23; *p* = 0.26; I²=64%; Supplementary Figure S8), pyrexia (OR 1.07; 95% CI 0.66–1.75; *p* = 0.78; I²=51%; Supplementary Figure S9), aspartate aminotransferase increased (OR 0.91; 95% CI 0.69–1.21; *p* = 0.53; I²=0%; Supplementary Figure S10), alanine aminotransferase increased (OR 0.78; 95% CI 0.57–1.06; *p* = 0.11; I²=0%; Supplementary Figure S11), or thrombocytopenia (OR 0.82; 95% CI 0.51–1.33; *p* = 0.43; I²=44%; Supplementary Figure S12).

In addition, Zolbetuximab plus chemotherapy significantly increased grade ≥ 3 of all treatment-emergent events (OR 1.44; 95% CI 1.09–1.90; *p* = 0.01; I²=8%; Supplementary Figure S13), nausea (OR 2.78; 95% CI 1.76–4.40; *p* < 0.01; I²=0%; Supplementary Figure S14), vomiting (OR 3.33; 95% CI 2.13–5.19; *p* < 0.01; I²=0%; Supplementary Figure S15), neutropenia (OR 1.55; 95% CI 1.08–2.22; *p* = 0.02; I²=14%; Supplementary Figure S16), asthenia (OR 2.48; 95% CI 1.24–4.95; *p* = 0.01; I²=0%; Supplementary Figure S17), and weight decrease (OR 2.69; 95% CI 1.02–7.15; *p* = 0.05; I²=0%; Supplementary Figure S18). There was no significant difference between groups for grade ≥ 3 of decrese appetite (OR 2.07; 95% CI 0.71–6.04; *p* = 0.18; I²=49%; Supplementary Figure S19), diarrhea (OR 1.00; 95% CI 0.59–1.68; *p* = 0.99; I²=0%; Supplementary Figure S20), anaemia (OR 1.00; 95% CI 0.68–1.46; *p* = 0.99; I²=0%; Supplementary Figure S21), fatigue (OR 1.13; 95% CI 0.65–1.94; *p* = 0.67; I²=0%; Supplementary Figure S22), abdominal pain (OR 0.88; 95% CI 0.22–3.48; *p* = 0.86; I²=44%; Supplementary Figure S23), pyrexia (OR 1.59; 95% CI 0.19–12.95; *p* = 0.67; I²=0%; Supplementary Figure S24), oedema peripheral (OR 3.90; 95% CI 0.43–35.44; *p* = 0.23; I²=0%; Supplementary Figure S25), aspartate aminotransferase increased (OR 0.79; 95% CI 0.36–1.73; *p* = 0.56; I²=0%; Supplementary Figure S26), alanine aminotransferase increased (OR 0.41; 95% CI 0.14–1.25; *p* = 0.12; I²=14%; Supplementary Figure S27), and thrombocytopenia (OR 0.86; 95% CI 0.38–1.93; *p* = 0.71; I²=0%; Supplementary Figure S28).

### Sensitivity analyses and risk of bias

We performed leave-one-out sensitivity analyses for all outcomes. There was no significant difference in emergency events grade ≥ 3 omitting FAST 2021 or SPOTLIGHT 2023. There was a significant difference favoring the control group in decreasing appetite in both the general and the subgroup populations omitting FAST 2021. There was a significant difference favoring the control group in neutropenia in both the general and subgroup populations omitting SPOTLIGHT 2023. There was no significant difference in asthenia grade ≥ 3 omitting SPOTLIGHT 2023. There was a significant difference in favor of the intervention group for abdominal pain omitting FAST 2021. There was a significant difference in favor of the control group for weight decreased grade ≥ 3 omitting GLOW 2023. There was a significant difference in favor of the intervention group for ALT levels grade ≥ 3 FAST 2021. Leave-one-out sensitivity analysis of the main outcomes is detailed in Supplementary Figure S29.

Risk of individual within-study bias is represented in the Rob 2 traffic-light diagram (Supplementary Figure S30). All studies were considered low risk of bias.

## Discussion

In this systematic review and meta-analysis involving 3 studies and 1,233 patients, we compared Zolbetuximab plus chermotherapy against chermotherapy only for patients with primary advanced or recurrent GC/GEJ/EC. The main findings from the pooled analyses were as follows: (1) PFS was improved in patients receiving Zolbetuximab; (2) OS showed a significant difference favoring Zolbetuximab; and (3) adverse events, particularly nausea and vomiting of any grade and grade 3 or more, increased in the Zolbetuzimab group.

Zolbetuximab (IMAB363) is a chimeric IgG1 monoclonal antibody with a high affinity for CLDN 18.2 present on the cell surface, which induces cell death due to antibody-dependent cellular cytotoxicity and complement-dependent cytotoxicity [23, 24, 38–41]. It was granted a priority review by the Food and Drug Administration (FDA) in 2023 for patients with locally advanced, inoperable or metastatic GC/GEJ with moderate to strong expression of CLDN18.2, HER2 negative GC or GEJ adenocarcinoma [42, 43].

A phase III study, CheckMate-649, showed that nivolumab improved PFS (HR 0.68; 98% CI 0.56–0.81; *p* < 0.0001) compared to chemotherapy with fluorouracil, leucovorin, and oxaliplatin (FOLFOX) or capecitabine and oxaliplatin (XELOX) for GC/GEJ cancer [16]. Additionally, the phase III ToGA study showed that trastuzumab plus chemotherapy improved PFS (HR 0.71; 95% CI 0.59–0.85; *p* < 0.0002) compared to chemotherapy alone in patients with HER2-positive, GC or GEJ cancer [44]. Therefore, similar to these studies, our meta-analysis shows a significant benefit with the addition of Zolbetuximab to chemotherapy (HR 0.64; 95% CI 0.49–0.84; *p* < 0.01) in patients with GC/GEJ cancer.

Regarding OS, our results show statistical significance in favor of the Zolbetuximab group for patients with CLDN18.2-positive, HER2-negative GC/GEJ cancer (HR 0.72; 95% CI 0.62–0.83; *p* < 0.01). Similarly, the addition of other targeted therapies to cytotoxic chemotherapy for GC/GEJ cancer also represents superior benefit over chemotherapy alone, such as anti-HER2 (HR 0.74; 95% CI 0.60–0.91; *p* < 0.0046) [44] and anti-PD1 (HR 0.71; 98.4% CI 0.59–0.86; *p* < 0.0001) [16] for advanced or metastatic GC/GEJ cancer.

In the analysis of the studies, the median PFS was higher than the chemotherapy-only group in all studies, with 7.5 months in Zolbetuximab plus EOX (Epirubicin, Oxaliplatin and Capecitabine) and 5.3 months with EOX in the FAST trial [35]; 8.21 versus 6.80 months with placebo plus CAPOX in the GLOW trial [36] and 10.61 in the Zolbetuximab plus mFOLFOX6 group versus 8.67 months in placebo plus mFOLFOX6 group in SPOTLIGHT trial [37]. Likewise, a benefit of increased OS was observed for all studies, with 13.0 versus 8.3 months in the NCT01630083 [35]; 14.39 versus 12.16 months in the NCT03653507 [36] and, NCT03504397 [37], 18.23 versus 15.54 months.

Although our meta-analysis shows no benefit for ORR (*p* = 0.34), DCR (*p* = 0.68), PR (*p* = 0.95) and SD (*p* = 0.09) for patients in the Zolbetuximab group, we found a positive association for CR (*p* = 0.01) and PD (*p* = 0.04). These data are similar to those found in the phase III JACOB study (NCT 01774786), which showed that the addition of Pertuzumab to Trastuzumab and chemotherapy resulted in CR for 5.7% (20) versus 2%(7) of the placebo group and a decrease in the incidence of PR, with 4.8% (17) versus 8.2% (29), for HER2-positive GC/GEJ cancer patients [45].

Adverse events on overall well-being associated with the chosen pharmacotherapy generally have a detrimental influence on the patient’s daily life, compromising their routine activities and emotional state. Although the frequency of adverse events is usually higher in combined chemotherapy, only nausea, vomiting, decreased appetite, peripheral edema, neutropenia, asthenia and decreased weight showed a statistically significant difference between the groups, both of which tended to favor chemotherapy alone [23, 24]. However, considering the overall benefit achieved with the addition of Zolbetuximab, its clinical use should be considered. Additionally, it is important to note that the included studies did not use steroids as prophylaxis for nausea and vomiting, due to the sponsor concern that they would abrogate the effect of Zolbetuximab.

This study has some limitations. Firstly, the analysis was based on a restricted (limited) number of RCTs. However, the pooled analysis of most of the results suggests that our meta-analysis conveys the best available evidence for the use of Zolbetuximab plus chemotherapy as a treatment for CLD18.2 positive, GC/GEJ adenocarcinoma.

## Conclusion

This is the first meta-analysis of RCTs to evaluate first-line with Zolbetuximab for advanced CLDN18.2-positive gastric or gastro-oesophageal adenocarcinoma. Our results support that the addition of Zolbetuximab to chemotherapy is associated with significant improvement in PFS and OS. The combination is not associated with increased toxicities to the treatment.

### Electronic supplementary material

Below is the link to the electronic supplementary material.

Supplementary Material 1

## Data Availability

All data generated and/or analysed during this study are included in this published article [and its supplementary information files]. Requests for materials should be addressed to F.C.A.M.; francisco.cezar2205@gmail.com.

## Abbreviations

Cls: Confidence intervals
GC: Gastric Adenocarcinoma
CLDN18.2: Claudin-18 isoform 2
CR: Complete Response
GEJ: Adenocarcinomas of the gastro-oesophageal junction
HER2: Human epidermal growth factor recptor 2
HRs: Hazard ratio
OR: Odds ratio
ORR: Objective Response Rate
OS: Overall survival
RCTs: Randomized controlled trials
SD: Stable Disease
PD: Progressive Disease
PD-L1: Programmed death-ligand 1
PFS: Progression-free survival
PR: Partial Response
