# Supplementary material for: Efficacy and safety of Zolbetuximab plus chemotherapy for advanced CLDN18.2-positive gastric or gastro-oesophageal adenocarcinoma: a meta-analysis of randomized clinical trials
Source: BMC Cancer. 2024 Feb 21;24:240. doi: 10.1186/s12885-024-11980-w (PMC10882870; doi:10.1186/s12885-024-11980-w)

**Efficacy And Safety Of Zolbetuximab Plus Chemotherapy For Advanced CLDN18.2-positive Gastric Or Gastro-oesophageal Adenocarcinoma: A Meta-analysis Of Randomized Clinical Trials.**

Francisco Cezar Aquino de Moraes^1*^, Eric Pasqualotto^2^, Matheus Pedrotti Chavez^2^, Rafael Oliva Morgado Ferreira^2^, Tiago Biachi De Castria^3,4^, Rommel Mario Rodríguez Burbano^5^.

^1^ Federal University of Pará, Belém, Pará, 66073-005, Brazil

^2^ Federal University of Santa Catarina, Florianópolis, Santa Catarina, 88040-900, Brazil;

^3^ Departament of Gastrointestinal Oncology, Moffitt Cancer Center, 12902 USF Magnolia Drive, Tampa, FL 33612, USA

^4^ Morsani College of Medicine, University of South Florida, 12901 Bruce B. Downs Blvd., Tampa, FL 33612, USA

^5^ Ophir Loyola Hospital, 66063-240, Belém, PA, Brazil

**Supplementary Material**

**Supplementary Table 1.** Inclusion and exclusion criteria of included studies.

**Supplementary Table 2.** Search strategies.

**Supplementary Table 3.** Additional baseline characteristics of included studies

**Supplementary Figure S1.** Any grade of all treatment-emergent events.

**Supplementary Figure S2.** Any grade of diarrhea.

**Supplementary Figure S3.** Any grade of neutropenia.

**Supplementary Figure S4.** Any grade of anemia.

**Supplementary Figure S5.** Any grade of fatigue.

**Supplementary Figure S6.** Any grade of asthenia.

**Supplementary Figure S7.** Any grade of abdominal pain.

**Supplementary Figure S8.** Any grade of weight decrease.

**Supplementary Figure S9.** Any grade of pyrexia.

**Supplementary Figure S10.** Any grade of aspartate aminotransferase increased.

**Supplementary Figure S11.** Any grade of alanine aminotransferase increased.

**Supplementary Figure S12.** Any grade of thrombocytopenia.

**Supplementary Figure S13.** Grade ≥3 of all treatment-emergent events.

**Supplementary Figure S14.** Grade ≥3 of nausea.

**Supplementary Figure S15.** Grade ≥3 of vomiting.

**Supplementary Figure S16.** Grade ≥3 of neutropenia.

**Supplementary Figure S17.** Grade ≥3 of asthenia.

**Supplementary Figure S18.** Grade ≥3 of weight decrease.

**Supplementary Figure S19.** Grade ≥3 of decrease appetite.

**Supplementary Figure S20.** Grade ≥3 of diarrhea.

**Supplementary Figure S21.** Grade ≥3 of anemia.

**Supplementary Figure S22.** Grade ≥3 of fatigue.

**Supplementary Figure S23.** Grade ≥3 of abdominal pain.

**Supplementary Figure S24.** Grade ≥3 of pyrexia.

**Supplementary Figure S25.** Grade ≥3 of oedema peripheral.

**Supplementary Figure S26.** Grade ≥3 of aspartate aminotransferase increased.

**Supplementary Figure S27.** Grade ≥3 of alanine aminotransferase increased.

**Supplementary Figure S28.** Grade ≥3 of thrombocytopenia.

**Supplementary Figure S29.** Leave-one-out sensitivity analyses. **A.** Progression-free survival. **B.** Overall survival.

**Supplementary Figure S30.** Critical appraisal of RCTs according to the Cochrane Collaboration’s tool for assessing risk of bias in randomized trials.

**Supplementary Table S1.** Inclusion and exclusion criteria of included studies

| **Study** | **Inclusion Criteria** | **Exclusion Criteria** |
| --- | --- | --- |
| FAST (NCT01630083) | 1. Histologically confirmed adenocarcinoma of the stomach, the oesophagus, or the gastro-oesophageal junction.  2. Inoperable locally advanced disease, resections or resections with resection margin outcome, or recurrent or metastatic disease. After receiving treatment cycles 2 and 4 of this study protocol, patients were re-evaluated for resectability according to institutional guidelines.  3. CLDN18.2 expression confirmed by immunohistochemistry in a paraffin-embedded tumour tissue sample. Any tumour with a staining intensity of 2+ or 3+ (the sum was decisive) in ≥40% of the tumour cells.  4. Measurable and/or non-measurable disease as defined according to Response Evaluation Criteria in Solid Tumors v1.1.  5. Aged ≥18 years.  6. Written informed consent.  7. Eastern Cooperative Oncology Group performance status 0 to 1.  8. Life expectancy >3 months  9. HER2/neu–negative patients with HER2/neu–positive status, but not eligible for trastuzumab therapy by discretion of the investigator.  10. Adequate cardiac function. Formal measurement of left cardiac ejection fraction was ≥55%.  11. Adequate hepatic function; bilirubin | 1. Prior severe allergic reaction or intolerance to a monoclonal antibody, including humanised or chimeric antibodies.  2. Prior severe allergic reaction or intolerance to the chemotherapeutics used in this study or any excipient in the respective formulations.  3. Previous chemotherapy for advanced disease.  4. Previous perioperative chemotherapy with curative intention within 6 months of the start of study treatment. If the interval was longer than 6 months (counted from stop date of the perioperative chemotherapy), patients were allowed in the study.  5. Radiotherapy within 4 weeks of start of study treatment (day 1 of cycle 1; 2-week interval was allowed if palliative radiotherapy was given to bone metastatic side peripherally and the patient recovered from acute toxicity).  6. Other investigational agents or devices used concurrently or within 4 weeks prior to this study (day 1 of cycle 1).  7. Known human immunodeficiency virus infection or known symptomatic hepatitis (A, B, and/or C).  8. Clinical symptoms of cerebral metastases.  9. Clinically significant (i.e., active) cardiac disease. History of myocardial infarction or hospitalization for congestive heart failure within 12 months of enrolment.  10. Other clinically significant disease or comorbidity which may have adversely affected the safe delivery of treatment within this study, including, but not limited to, any of the following: ongoing or active infection that required parenteral antibiotics, uncontrolled hypertension, present cardiac arrhythmia with serious hemodynamic consequences, or unstable angina pectoris.  11. Psychiatric illness or social situations that would preclude study compliance.  12. Pregnancy or breastfeeding.  13. Gastric bleeding within the last 2 weeks; symptomatic peptic ulcer.  14. Concurrent systemic immunosuppressive therapy, in particular systemic corticoids, were to be stopped 2 weeks prior to therapy (day 1 of cycle 1). Inhaled and topically applied steroids were the exception and were allowed. Systemic steroids were to be avoided as long as the patient was being treated with study medication. An exception was in the case of uncontrollable nausea and/or vomiting.  15. Previous treatments with maximum cumulative doses of epirubicin >500 mg/m2 and/or other anthracyclines and anthracenediones.  16. Treatment with sorivudine or analogs.  17. Known peripheral neuropathy greater than grade 1 (absence of deep tendon reflexes as the sole neurological abnormality did not render the patient ineligible).  18. Malabsorption syndrome or inability to take oral medication (administration of capecitabine by nasogastric or jejunostomy feeding tube was permitted).  19. Known dihydropyrimidine dehydrogenase deficiency.  20. History of interstitial lung disease (e.g., pneumonitis or pulmonary fibrosis) or evidence of interstitial lung disease on baseline chest CT scan only if clinically relevant.  21. Evidence of dyspnoea at rest.  22. Prior or current active autoimmune disease that required management with immunosuppression. This included inflammatory bowel disease, systemic vasculitis, scleroderma, psoriasis, haemolytic anaemia, immune-mediated thrombocytopenia, rheumatoid arthritis, systemic lupus erythematosus, Sjogren's syndrome, sarcoidosis, or other rheumatologic disease. Asthma and chronic obstructive pulmonary disease that did not require daily systemic corticosteroids was acceptable.  23. Sinusoidal obstruction syndrome, formerly known as venoocclusive disease, if present, should have been stable or improving. |
| GLOW (NCT03653507) | 1. IRB/IEC-approved written informed consent and privacy language as per national regulations (for example, Health Insurance Portability and Accountability Act (HIPAA) authorization for US sites) must be obtained from the patient or legally authorized representative (if applicable) before any study-related procedures.  2. Patient is considered an adult (for example, ≥18 years of age in the USA) according to local regulation at the time of signing the informed consent.  3. A female patient is eligible to participate if she is not pregnant (negative serum pregnancy test at screening; female patients with elevated serum beta human chorionic gonadotropin (βhCG) and a demonstrated non-pregnant status through additional testing are eligible) and at least one of the following conditions applies.   - Not a woman of childbearing potential (WOCBP) as defined in Protocol Appendix 12.3 Contraception Requirements - OR - WOCBP who agrees to follow the contraceptive guidance as defined in Protocol Appendix 12.3 Contraception Requirements throughout the treatment period and for 9 months after the final administration of oxaliplatin and 6 months after the final administration of all other study drugs   4. Female patient must agree not to breastfeed starting at screening and throughout the study period and for 6 months after the final study treatment administration.  5. Female patient must not donate ova starting at screening and throughout the study period and for 9 months after the final administration of oxaliplatin and for 6 months after the final administration of all other study drugs.  6. Male patient with female partner(s) of childbearing potential must agree to use contraception as detailed in Protocol Appendix 12.3 Contraception Requirements during the treatment period and for 6 months after the final study treatment administration.  7. Male patient must not donate sperm during the treatment period and for 6 months after the final study treatment administration.  8. Male patient with a pregnant or breastfeeding partner(s) must agree to remain abstinent or use a condom for the duration of the pregnancy or time partner is breastfeeding throughout the study period and for 6 months after the final study treatment administration.  9. Patient agrees not to participate in another interventional study while receiving study drug in the present study.  10. Patient has histologically confirmed diagnosis of G/GEJ adenocarcinoma.  11. Patient has radiologically confirmed locally advanced unresectable or metastatic disease within 28 days before randomization.  12. Patient has radiologically evaluable disease (measurable and/or non-measurable) according to RECIST version 1.1, per local assessment, ≤28 days before randomization. For patients with only one evaluable lesion and prior radiotherapy ≤3 months before randomization, the lesion must either be outside the field of prior radiotherapy or have documented progression after radiation therapy.  13. Patient’s tumor expresses CLDN18.2 in ≥75% of tumor cells, demonstrating moderate-to-strong CLDN18 membranous staining as determined by central immunohistochemistry testing.  14. Patient has a HER2-negative tumor as determined by local or central testing on a G/GEJ tumor specimen.  15. Patient has a ECOG performance status score 0 or 1.  16. Patient has predicted life expectancy ≥12 weeks, in the opinion of the investigator.  17. Patient must meet all of the following criteria based on the centrally or locally analyzed laboratory tests collected within 14 days before randomization. In the case of multiple sample collections within this period, the most recent sample collection with available results should be used to determine eligibility.   - Hemoglobin ≥9 g dl^−1^. Patients requiring transfusions are eligible if they have a post-transfusion hemoglobin ≥9 g dl^−1^. - Absolute neutrophil count (ANC) ≥1.5 × 10^9^/l - Platelets ≥100 × 10^9^/l - Albumin ≥2.5 g dl^−1^ - Total bilirubin ≤1.5× upper limit of normal (ULN) without liver metastases (or <3.0× ULN if liver metastases are present) - Aspartate aminotransferase and alanine aminotransferase ≤2.5× ULN without liver metastases (or ≤5× ULN if liver metastases are present) - Estimated creatinine clearance ≥30 ml min^−1^ - Prothrombin time/international normalized ratio and partial thromboplastin time ≤1.5× ULN (except for patients receiving anti-coagulation therapy) | 1. Patient has received prior systemic chemotherapy for locally advanced unresectable or mG/GEJ adenocarcinoma. However, patient may have received either neoadjuvant or adjuvant chemotherapy, immunotherapy or other systemic anti-cancer therapies as long as it was completed at least 6 months before randomization.  2. Patient has received radiotherapy for locally advanced unresectable or mG/GEJ adenocarcinoma ≤14 days before randomization and has not recovered from any related toxicity.  3. Patient has received treatment with herbal medications or other treatments that have known anti-tumor activity within 28 days before randomization.  4. Patient has received systemic immunosuppressive therapy, including systemic corticosteroids, within 14 days before randomization. Patient using a physiologic replacement dose of hydrocortisone or its equivalent (defined as up to 30 mg per day of hydrocortisone or up to 10 mg per day of prednisone), receiving a single dose of systemic corticosteroids or receiving systemic corticosteroids as premedication for radiologic imaging contrast use is eligible.  5. Patient has received other investigational agents or devices within 28 days before randomization.  6. Patient has prior severe allergic reaction or intolerance to known ingredients of zolbetuximab or other monoclonal antibodies, including humanized or chimeric antibodies.  7. Patient has known immediate or delayed hypersensitivity, intolerance or contraindication to any component of study treatment.  8. Patient has prior severe allergic reaction or intolerance to any component of CAPOX.  9. Patient has known dihydropyrimidine dehydrogenase deficiency. (Note that screening for dihydropyrimidine dehydrogenase deficiency should be conducted per local requirements.)  10. Patient has a complete gastric outlet syndrome or a partial gastric outlet syndrome with persistent/recurrent vomiting.  11. Per investigator judgment, patient has significant gastric bleeding and/or untreated gastric ulcers that exclude the patient from participation.  12. Patient has a known history of a positive test for human immunodeficiency virus (HIV) infection or known active hepatitis B (HB; positive HB surface antigen (HBs Ag)) or hepatitis C infection. (Note: Screening for these infections should be conducted per local requirements.)   - For patients who are negative for HBs Ag but HB core antibody (HBc Ab) positive, an HB DNA test will be performed, and, if positive, the patient will be excluded. - Patients with positive hepatitis C virus (HCV) serology but negative HCV RNA test are eligible. - Patients treated for HCV with undetectable viral load results are eligible.   13. Patient has an active autoimmune disease that has required systemic treatment within the past 3 months before randomization.  14. Patient has an active infection requiring systemic therapy that has not completely resolved within 7 days before randomization.  15. Patient has significant cardiovascular disease, including any of the following.   - Congestive heart failure (defined as New York Heart Association class III or IV), myocardial infarction, unstable angina, coronary angioplasty, coronary stenting, coronary artery bypass graft, cerebrovascular accident or hypertensive crisis within 6 months before randomization - History of clinically significant ventricular arrhythmias (that is, sustained ventricular tachycardia, ventricular fibrillation or Torsades de Pointes) - QTc interval >450 ms for male patients; QTc interval >470 ms for female patients - History or family history of congenital long QT syndrome - Cardiac arrhythmias requiring anti-arrhythmic medications (patients with rate controlled atrial fibrillation for >1 month before randomization are eligible)   16. Patient has history of central nervous system (CNS) metastases and/or carcinomatous meningitis from G/GEJ cancer.  17. Patient has known peripheral sensory neuropathy grade >1 unless the absence of deep tendon reflexes is the sole neurological abnormality.  18. Patient has had a major surgical procedure ≤28 days before randomization.  19. Patient without complete recovery from a major surgical procedure ≤14 days before randomization.  20. Patient has psychiatric illness or social situations that would preclude study compliance, per investigator judgment.  21. Patient has another malignancy for which treatment is required, per investigator judgment.  22. Patient has any concurrent disease, infection or comorbid condition that interferes with the ability of the patient to participate in the study, which places the patient at undue risk or complicates the interpretation of data, in the opinion of the investigator. |
| SPOTLIGHT (NCT03504397) | 1. Institutional Review Board (IRB)/Independent Ethics Committee (IEC) approved written informed consent and privacy language as per national regulations (e.g., Health Insurance Portability and Accountability Act [HIPAA] Authorization for US sites) must be obtained from the subject or legally authorized representative (if applicable) prior to any study-related procedures.  2. Subject is considered an adult (e.g., ≥ 18 years of age in the US) according to local regulation at the time of signing the informed consent.  3. Subject agrees not to participate in another interventional study while on study treatment.  4. Female subject must either: Be of non-childbearing potential:  • postmenopausal (defined as at least 1 year without any menses for which there is no other obvious pathological or physiological cause) prior to Screening,  • or, documented surgically sterile (e.g., hysterectomy, bilateral salpingectomy, bilateral oophorectomy) Or, if of childbearing potential:  • agree not to try to become pregnant during the study and for 6 months after the final study treatment administration,  • and, have a negative serum pregnancy test at Screening, (note: subjects with elevated serum beta human chorionic gonadotropin (βhCG) and a demonstrated non-pregnant status through additional testing are eligible)  • and, if heterosexually active, agree to consistently use 1 form of highly effective birth control* starting at Screening and throughout the study period and for 6 months after the final study drug administration.  5. Female subject must agree not to breastfeed starting at Screening and throughout the study period, and for 6 months after the final study treatment administration.  6. Female subject must not donate ova starting at Screening and throughout the study period, and for 6 months after the final study drug administration.  7. A sexually active male subject with female partner(s) who are of childbearing potential is eligible if: • Agrees to use a male condom starting at screening and continue throughout study treatment and for 6 months after the final study drug administration. • If he has not had a vasectomy or is not sterile as defined below, his female partner(s) is utilizing 1 form of highly effective birth control*starting at Screening and continue throughout study treatment and for 6 months after the he receives his final study drug administration.  8. Male subject must not donate sperm starting at Screening and throughout the study period and for 6 months after the final study drug administration.  9. Male subject with a pregnant or breastfeeding partner(s) must agree to remain abstinent or use a condom for the duration of the pregnancy or for the time partner is breastfeeding throughout the study period and for 6 months after the final study drug administration.  • Consistent and correct usage of established hormonal contraceptives that inhibit ovulation  • Established intrauterine device (IUD) or intrauterine system (IUS)  • Bilateral tubal occlusion  • Vasectomy (a vasectomy is a highly effective contraception method provided the absence of sperm has been confirmed. If not, an additional highly effective method of contraception should be used)  • Male is sterile due to a bilateral orchiectomy  • Sexual abstinence is considered a highly effective method only if defined as refraining from heterosexual activity during the entire period of risk associated with the study drug. The reliability of sexual abstinence needs to be evaluated in relation to the duration of the study and the preferred and usual lifestyle of the participant.  10. Subject has histologically confirmed diagnosis of Gastric or GEJ adenocarcinoma.  11. Subject has radiologically confirmed locally advanced unresectable or metastatic disease within 28 days prior to the first dose of study treatment.  12. Subject has measurable disease according to RECIST 1.1 within 28 days prior to the first dose of study treatment. For subjects with only 1 measurable lesion and prior radiotherapy, the lesion must be outside the field of prior radiotherapy or must have documented progression following radiation therapy.  13. Subject’s tumor expresses CLDN18.2 in ≥ 75% of tumor cells demonstrating moderate to strong membranous staining as determined by central IHC testing.  14. Subject has a HER2-Negative tumor as determined by local or central testing on a gastric or GEJ tumor specimen. Physical or Laboratory Findings  15. Subject has ECOG performance status 0 to 1.  16. Subject has predicted life expectancy ≥ 12 weeks in the opinion of the investigator.  17. Subject must meet all of the following criteria based on the centrally analyzed laboratory tests within 14 days prior to the first dose of study treatment. In case of multiple central laboratory data within this period, the most recent data should be used to determine eligibility.  • Hemoglobin (Hgb) ≥ 9 g/dl. NOTE: Subject must not have received any growth factor or blood transfusions within 14 days prior to the hematology values obtained at screening. Subjects requiring transfusions to meet eligibility criteria are not eligible.  • Absolute neutrophil count ≥ 1.5 x 109 /L  • Platelets ≥ 100 x 109 /L  • Albumin ≥ 2.5 g/dL  • Total bilirubin < 1.5 x upper limit of normal (ULN)  • Aspartate aminotransferase and alanine aminotransferase ≤ 2.5 x ULN without liver metastases (or ≤ 5 x ULN if liver metastases are present)  • Either serum creatinine ≤ 1.5 x ULN or estimated glomerular filtration rate ≥ 45 mL/min/1.73 m 2  • Prothrombin time/international normalized ratio and partial thromboplastin time ≤ 1.5 x ULN (except for subjects receiving anticoagulation therapy) | 1. Subject has received prior systemic chemotherapy for locally advanced unresectable or metastatic gastric or GEJ adenocarcinoma. However, subject may have received either neoadjuvant or adjuvant chemotherapy as long as it was completed at least 6 months prior to the first dose of study treatment.  2. Subject has received radiotherapy for locally advanced unresectable or metastatic gastric or GEJ adenocarcinoma unless the radiotherapy was completed within 28 days prior to start of study treatment. Subject who received palliative radiotherapy to peripheral bone metastases ≥ 14 days prior to start of study treatment and has recovered from all acute toxicities is allowed.  3. Subject has received systemic immunosuppressive therapy, including systemic corticosteroids within 14 days prior to first dose of study treatment. Subjects using a physiologic replacement dose of hydrocortisone or its equivalent (defined as up to 30 mg per day of hydrocortisone or up to 10 mg per day of prednisone) are allowed.  4. Subject has received other investigational agents or devices within 28 days prior to first dose of study treatment. Medical History or Concurrent Disease  5. Subject has prior severe allergic reaction or intolerance to a monoclonal antibody, including humanized or chimeric antibodies.  6. Subject has known immediate or delayed hypersensitivity, intolerance or contraindication to any component of study treatment.  7. Subject has prior severe allergic reaction or intolerance to any component of mFOLFOX6.  8. Subject has known dihydropyrimidine dehydrogenase deficiency.  9. Subject has gastric outlet syndrome or persistent/recurrent vomiting.  10. Subject with recent gastric bleeding or symptomatic subjects with proven gastric ulcers that would exclude the subject from participation per investigator judgment.  11. Subject has a known history of a positive test for human immunodeficiency virus (HIV) infection or known active hepatitis B (positive HBs Ag) or C infection. For subjects who are negative for HBs Ag, but HBc Ab positive, an HB DNA test will be performed and if positive the subject will be excluded. Subjects with positive serology but negative HCV RNA test results are eligible.  12. Subject has an active autoimmune disease that has required systemic treatment within the past 2 years.  13. Subject has active infection requiring systemic therapy that has not completely resolved within 14 days prior to start of study treatment.  14. Subject has significant cardiovascular disease, including:  • Congestive heart failure (defined as New York Heart Association Class III or IV), myocardial infarction, unstable angina, coronary angioplasty, stenting, coronary artery bypass graft, cerebrovascular accident or hypertensive crisis within 6 months prior to administration of first dose of study drug.  • History of clinically significant ventricular arrhythmias (i.e., sustained ventricular tachycardia, ventricular fibrillation or Torsades de Pointes);  • QTc interval > 450 msec  • Cardiac arrhythmias requiring anti-arrhythmic medications (Subject with rate controlled atrial fibrillation for > 1 month prior to firdt dose of study drugs are eligible)  15. Subject has known active central nervous system metastases and/or carcinomatous meningitis.  16. Subject has know peripheral sensory neuropathy > Grade 1 unless the absence of deep tendon reflexes is the sole neurological abnormality.  17. Subject has hand a major surgical procedure and has not completely recovered within 28 days prior to the start of study treatment.  18. Subject has psychiatric illness or social situations that would preclude study compliance, per investigator judgment.  19. Subject has another malignancy for which treatment is required per investigator’s clinical judgment. |

**Supplementary Table S2.** Search Strategies

| **Database** | **Search Strategy** |
| --- | --- |
| **PubMed** | ((((((("Neoplasm, Stomach") OR ("Stomach Neoplasm") OR ("Neoplasms, Stomach") OR ("Gastric Neoplasms") OR ("Gastric Neoplasm") OR ("Neoplasm, Gastric") OR ("Neoplasms, Gastric") OR ("Cancer of Stomach") OR ("Stomach Cancers") OR ("Gastric Cancer") OR ("Cancer, Gastric") OR ("Cancers, Gastric") OR ("Gastric Cancers") OR ("Stomach Cancer") OR ("Cancer, Stomach") OR ("Cancers, Stomach") OR ("Cancer of the Stomach") OR ("Adenocarcinoma") OR ("Esophageal Neoplasms") OR ("Adenocarcinoma Of Esophagus") OR ("Junction, Esophagogastric") OR ("Gastroesophageal Junction") OR ("Gastroesophageal Junctions") OR ("Junction, Gastroesophageal") OR ("Junctions, Gastroesophageal") OR ("Esophagogastric Junction") AND ("IMAB362") OR ("IMAB-362") OR ("zolbetuximab") OR ("CLDN18 protein, human") OR ("Claudins") OR ("claudin-18 protein, human") OR ("claudin18 protein, human") OR ("claudin 18 protein, human") |
| **Embase** | ("Neoplasm, Stomach" OR "Stomach Neoplasm" OR "Neoplasms, Stomach" OR "Gastric Neoplasms" OR "Gastric Neoplasm" OR "Neoplasm, Gastric" OR "Neoplasms, Gastric" OR "Cancer of Stomach" OR "Stomach Cancers" OR "Gastric Cancer" OR "Cancer, Gastric" OR "Cancers, Gastric" OR "Gastric Cancers" OR "Stomach Cancer" OR "Cancer, Stomach" OR "Cancers, Stomach" OR "Cancer of the Stomach" OR "Adenocarcinoma" OR "Esophageal Neoplasms" OR "Adenocarcinoma Of Esophagus" OR "Junction, Esophagogastric" OR "Gastroesophageal Junction" OR "Gastroesophageal Junctions" OR "Junction, Gastroesophageal" OR "Junctions, Gastroesophageal" OR "Esophagogastric Junction") AND ("IMAB362" OR "IMAB-362" OR "zolbetuximab" OR "CLDN18 protein, human" OR "Claudins" OR "claudin-18 protein |
| **The Cochrane Library** | (((("Neoplasm, Stomach") OR ("Stomach Neoplasm") OR ("Neoplasms, Stomach") OR ("Gastric Neoplasms") OR ("Gastric Neoplasm") OR ("Neoplasm, Gastric") OR ("Neoplasms, Gastric") OR ("Cancer of Stomach") OR ("Stomach Cancers") OR ("Gastric Cancer") OR ("Cancer, Gastric") OR ("Cancers, Gastric") OR ("Gastric Cancers") OR ("Stomach Cancer") OR ("Cancer, Stomach") OR ("Cancers, Stomach") OR ("Cancer of the Stomach") OR ("Adenocarcinoma") OR ("Esophageal Neoplasms") OR ("Adenocarcinoma Of Esophagus") OR ("Junction, Esophagogastric") OR ("Gastroesophageal Junction") OR ("Gastroesophageal Junctions") OR ("Junction, Gastroesophageal") OR ("Junctions, Gastroesophageal") OR ("Esophagogastric Junction")):ti,ab,kw AND (("IMAB362") OR ("IMAB-362") OR ("zolbetuximab") OR ("CLDN18 protein, human") OR ("Claudins") OR ("claudin-18 protein, human") OR ("claudin18 protein, human") OR ("claudin 18 protein, human")):ti,ab,kw |

**Supplementary Table S3.** Additional baseline characteristics of included studies

| **Study** | **Location of lesions, n (%)** | | **ECOG performance status score, n (%)** | | **Previous gastrectomy, n (%)** | |
| --- | --- | --- | --- | --- | --- | --- |
|  | **IG** | **CG** | **IG** | **CG** | **IG** | **CG** |
| FAST 2021 | Liver 30 (39.0) Lung 17 (22.1) Lymph node 64 (83.1) Peritoneum 20 (26.0) Primary tumour 49 (63.6) Other 52 (67.5) | Liver 31 (36.9) Lung 14 (16.7) Lymph node 62 (73.8) Peritoneum 23 (27.4) Primary tumour 57 (67.9) Other 52 (61.9) | 0: 23 (29.9)  1: 54 (70.1) | 0: 25 (29.8)  1: 59 (70.2) | Yes: 82 (29%) No: 200 (71%) | Yes: 84 (30%) No: 199 (70%) |
| GLOW 2023 | NA | NA | 0: 108 (42.7) 1: 145 (57.3)† | 0: 108 (43.2) 1: 142 (56.8)† | Yes: 75 (29.5) No: 179 (70.5) | Yes: 75 (29.6) No: 178 (70.4) |
| SPOTLIGHT 2023 | Lymph node 101 (36) Peritoneum 94 (33) Liver 62 (22) Lung 36 (13) Bone 28 (10) Abdominal cavity 19 (7) Ovary 16 (6) | Lymph node 109 (39) Peritoneum 76 (27) Liver 75 (27) Lung 33 (12) Bone 23 (8) Abdominal cavity 17 (6) Ovary 19 (7) | 0: 125 (44) 1: 153 (54) 2: 1 (<1)*‡ | 0: 115 (41) 1: 163 (58) 2: 0‡ | Yes: 84 (30) No: 199 (70) | Yes: 82 (29) No: 200 (71) |

† Missing ECOG performance status score for 1 patient in Zolbetuximab plus CAPOX group and 3 patients in placebo plus CAPOX group. * The patient with ECOG performance status score of 2 had a score of 1 at screening and was thus eligible for enrolment. ‡ Missing ECOG performance status score for 4 patients in Zolbetuximab plus mFOLFOX6 group and 4 patients in placebo plus mFOLFOX6 group. CG, control group; ECOG, Eastern Cooperative Oncology Group; IG, intervention group; NA, not available.

**Supplementary Figure S1.** Any grade of all treatment-emergent events.

**
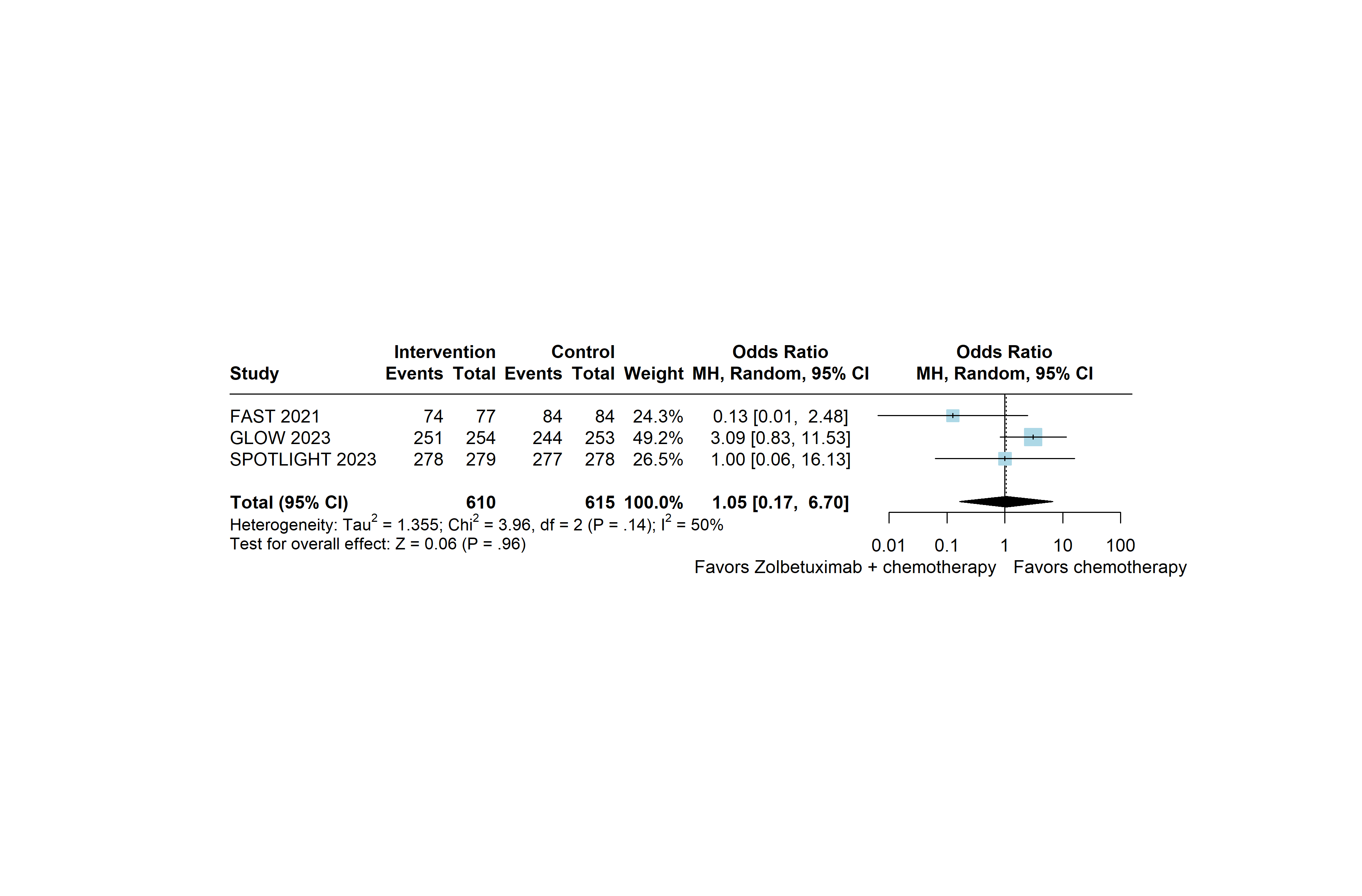
**

**Supplementary Figure S2.** Any grade of diarrhea.


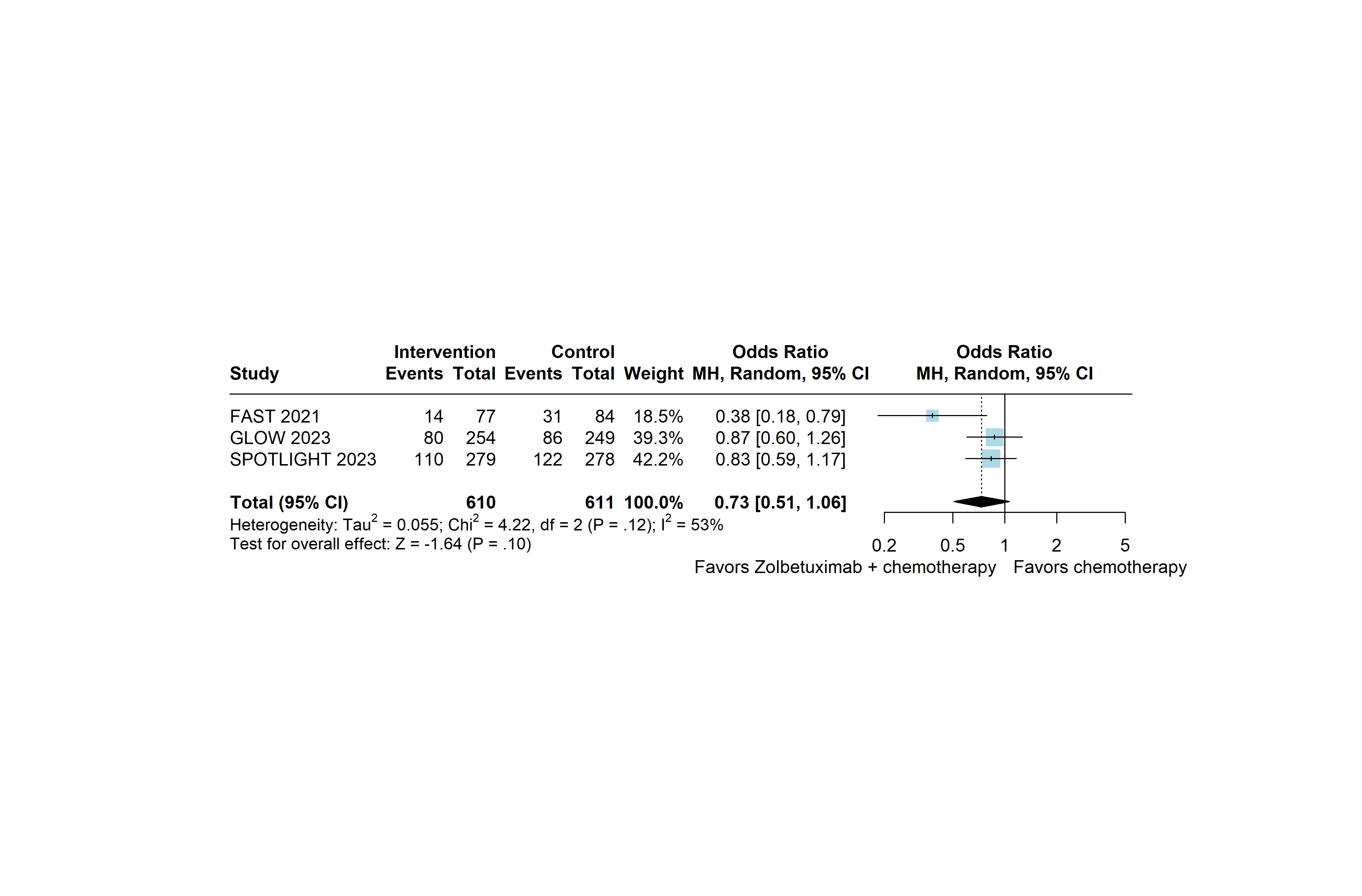


**Supplementary Figure S3.** Any grade of neutropenia.


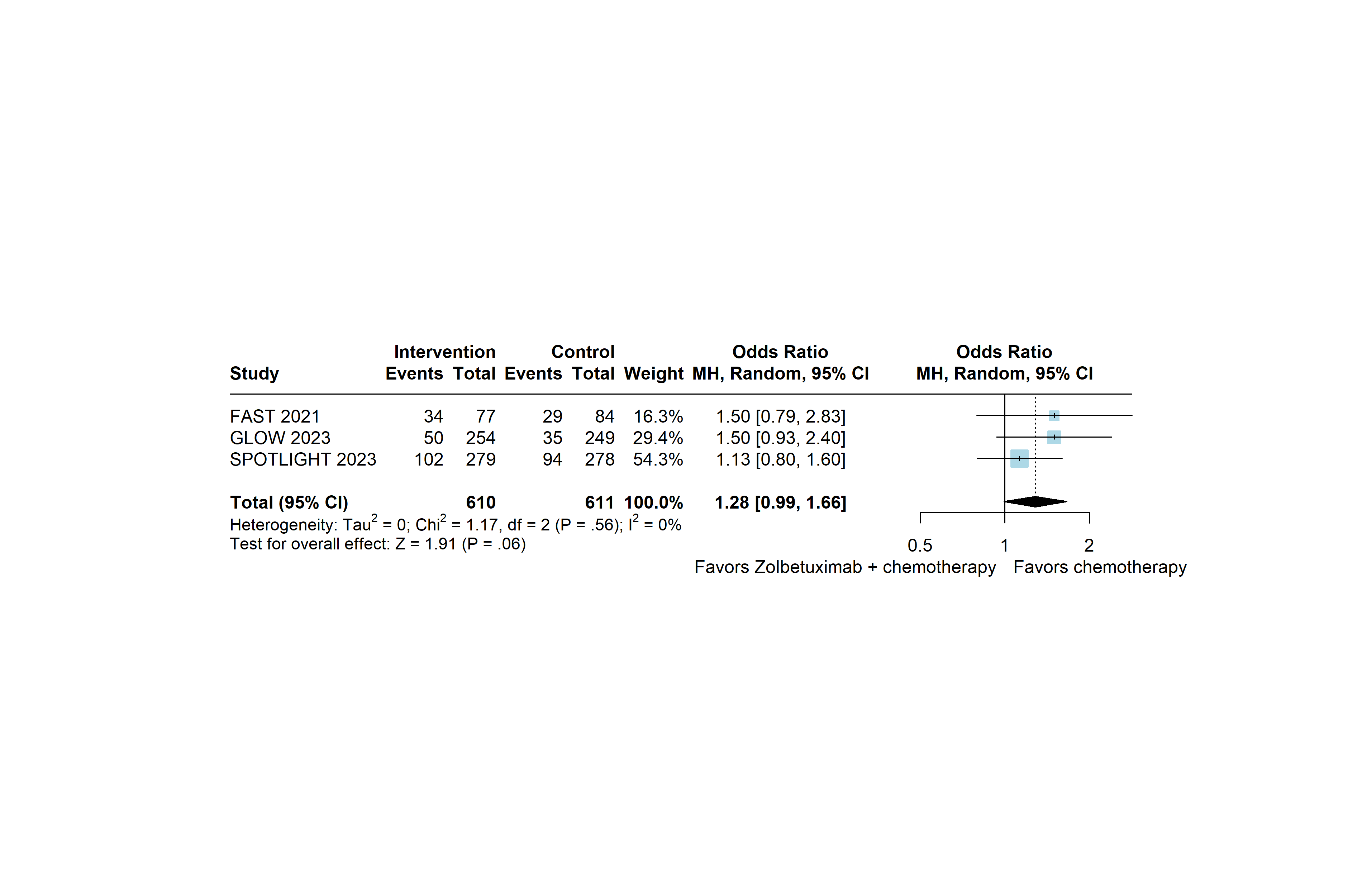


**Supplementary Figure S4.** Any grade of anemia.


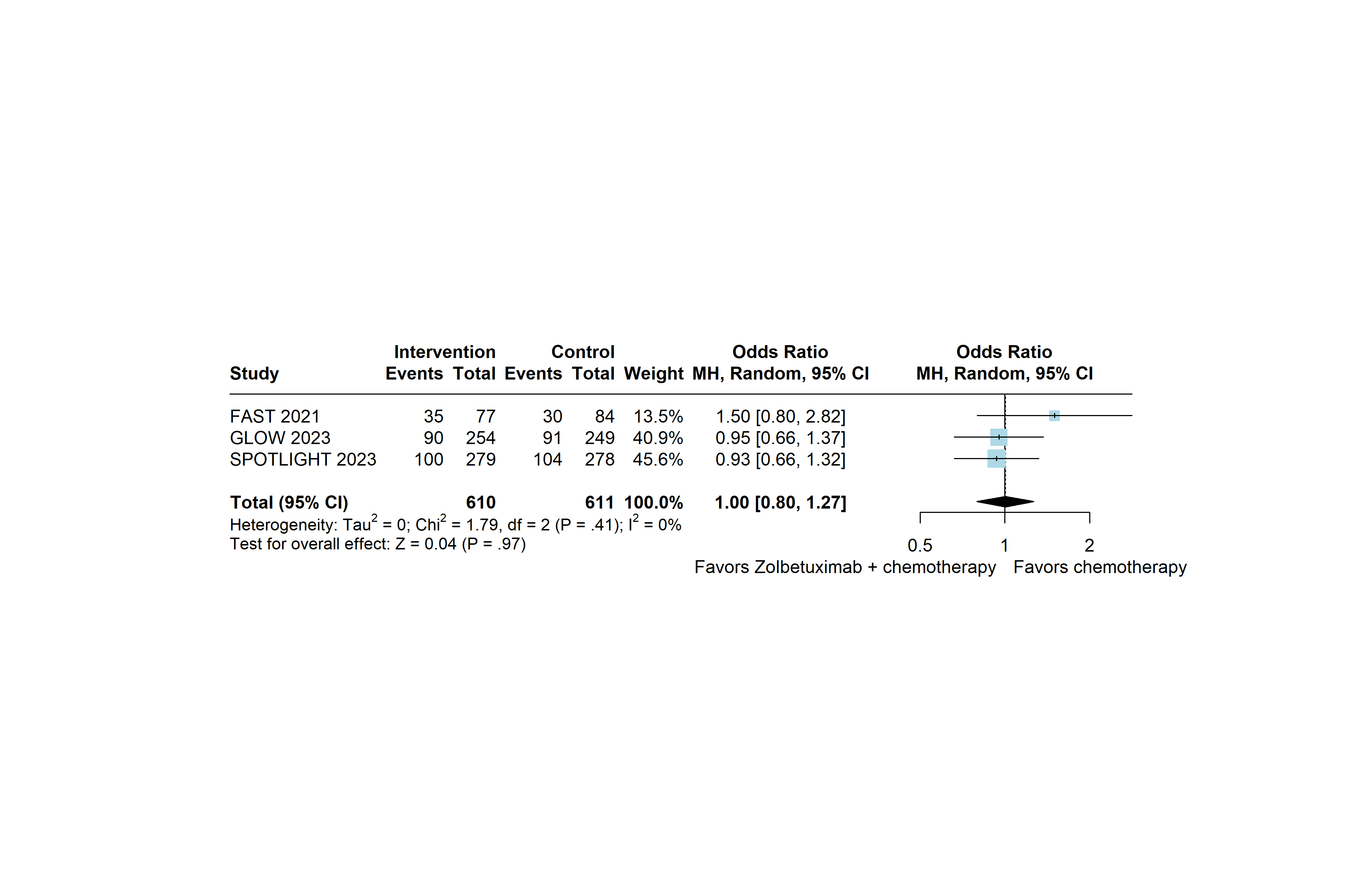


**Supplementary Figure S5.** Any grade of fatigue.


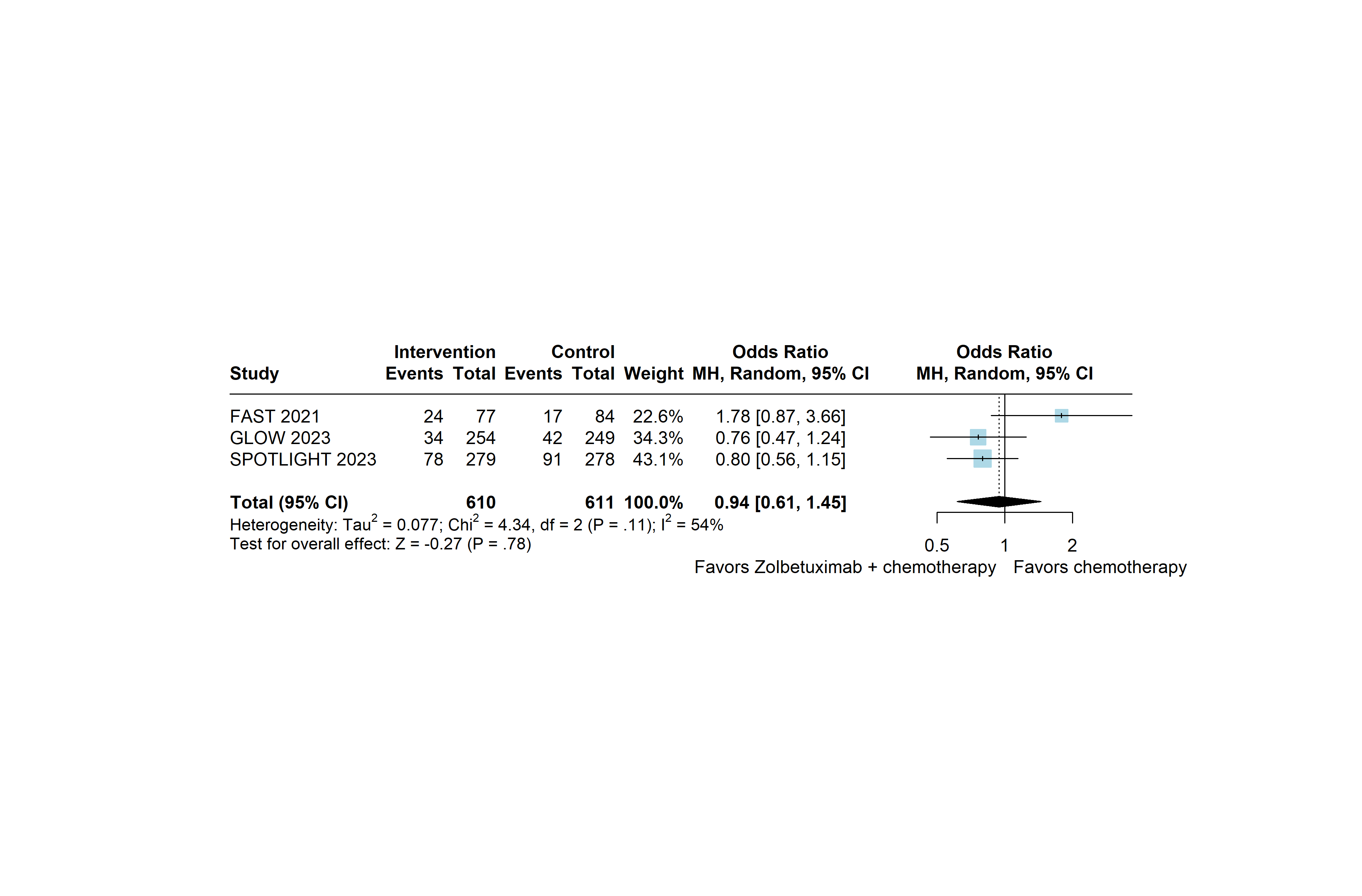


**Supplementary Figure S6.** Any grade of asthenia.


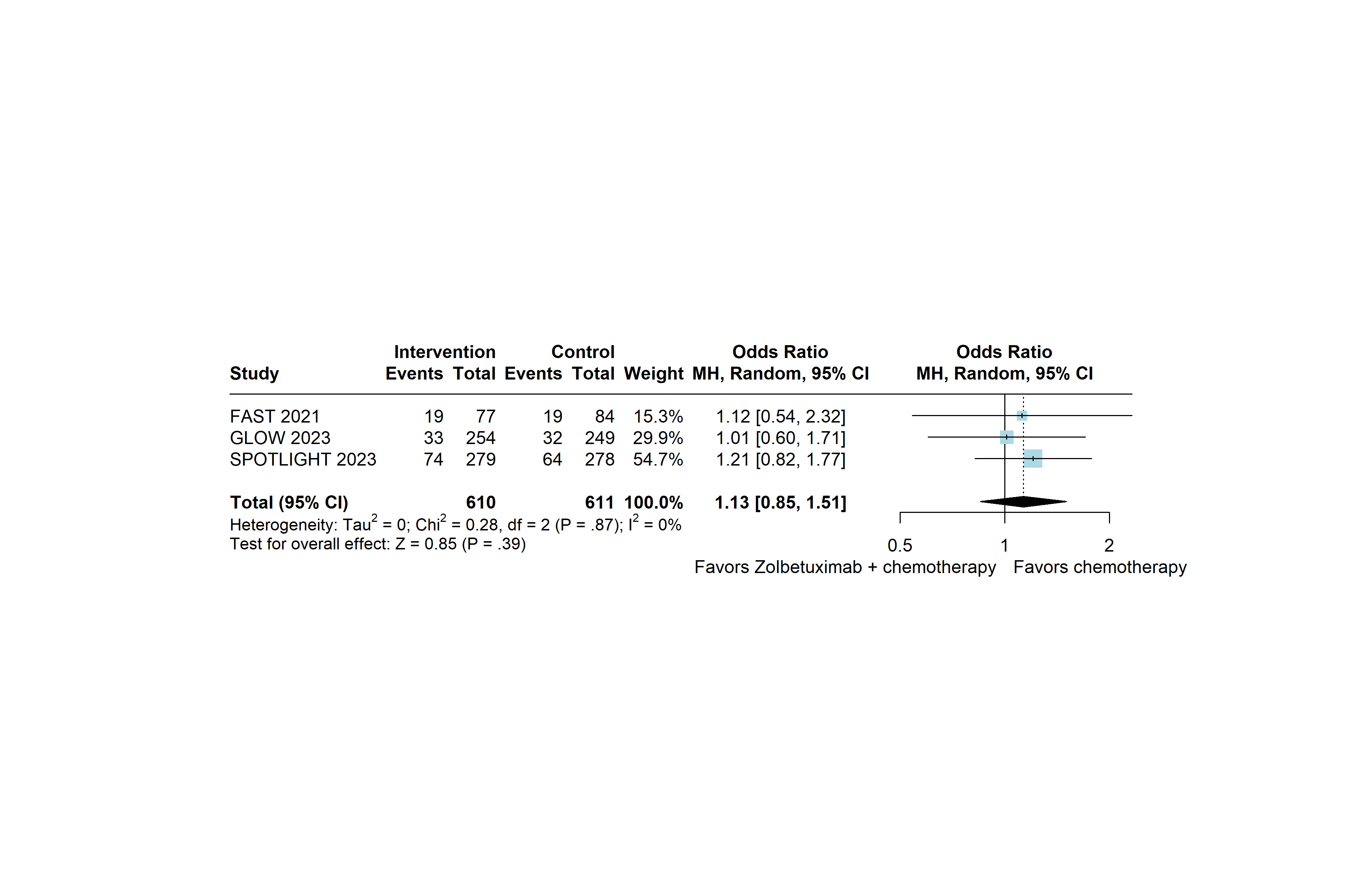


**Supplementary Figure S7.** Any grade of abdominal pain.


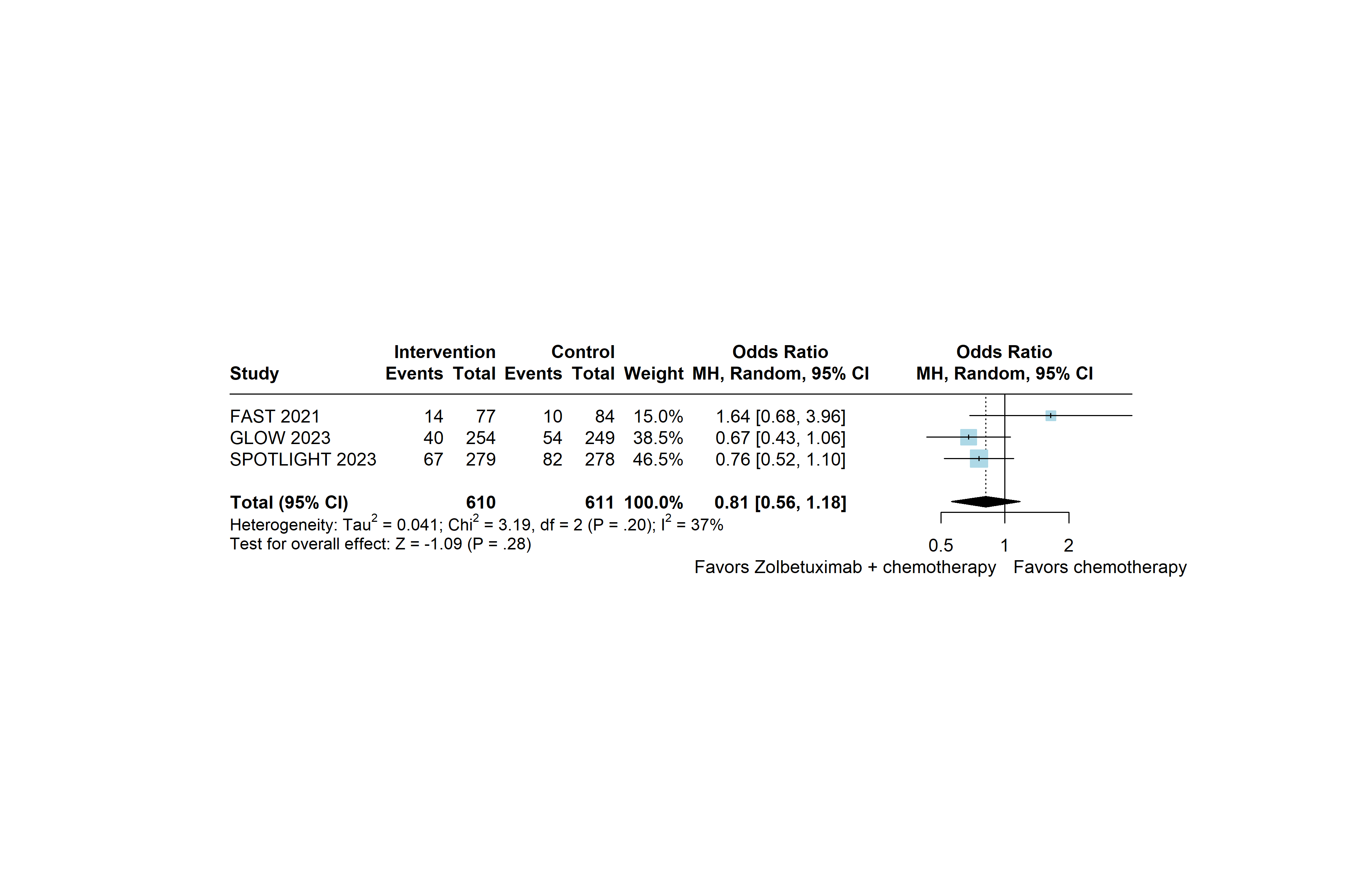


**Supplementary Figure S8.** Any grade of weight decrease.


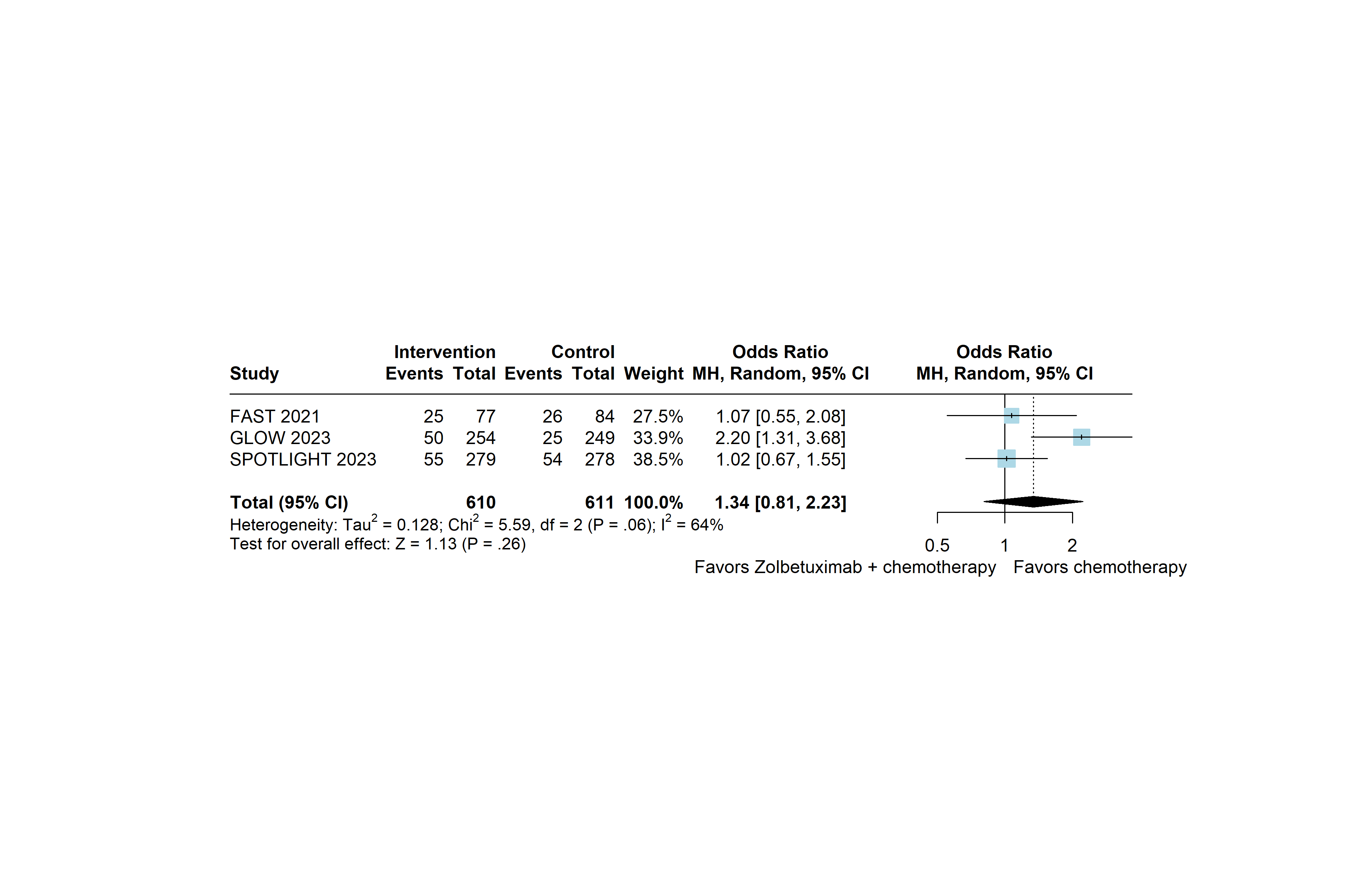


**Supplementary Figure S9.** Any grade of pyrexia.


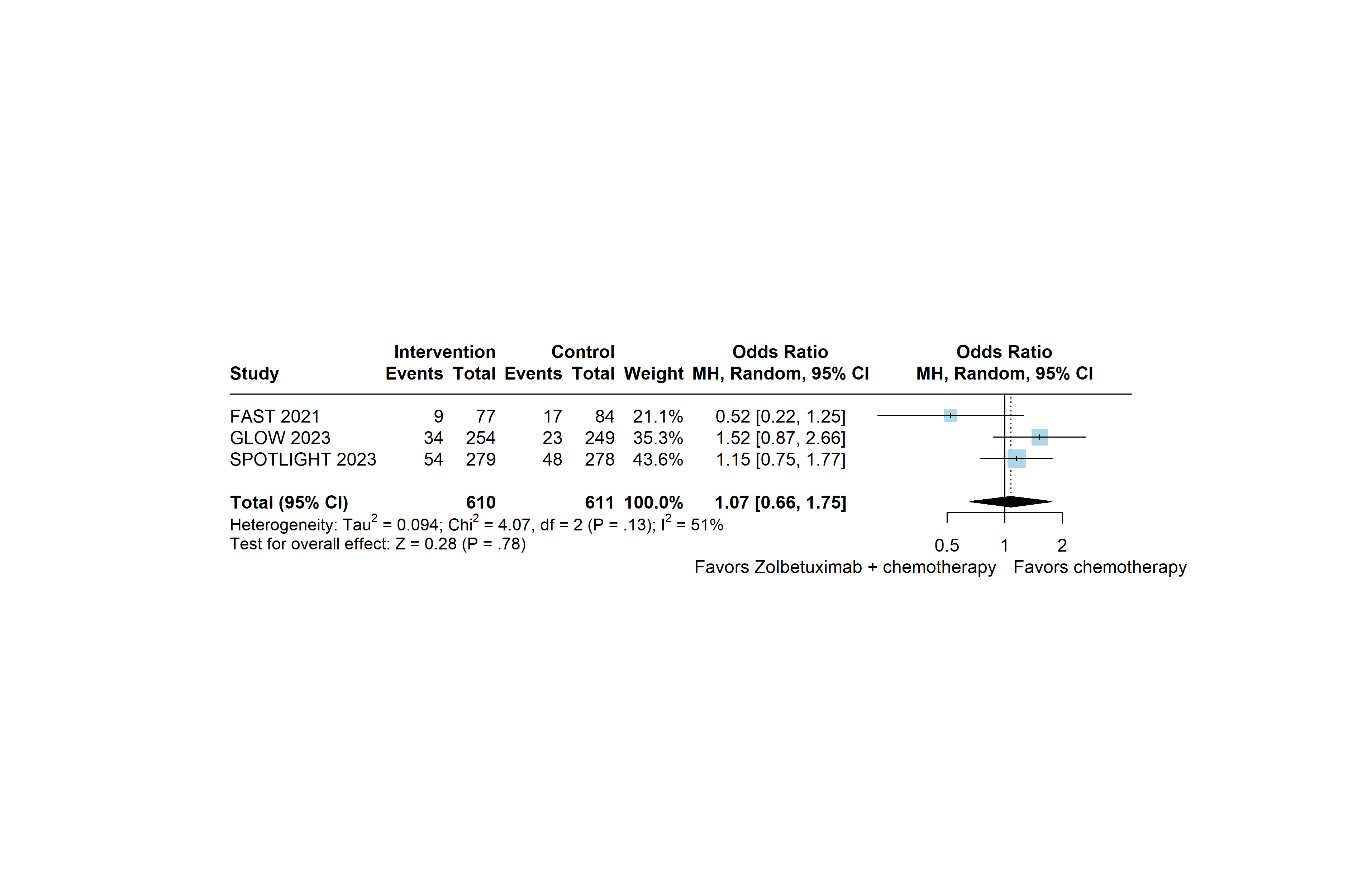


**Supplementary Figure S10.** Any grade of aspartate aminotransferase increased.


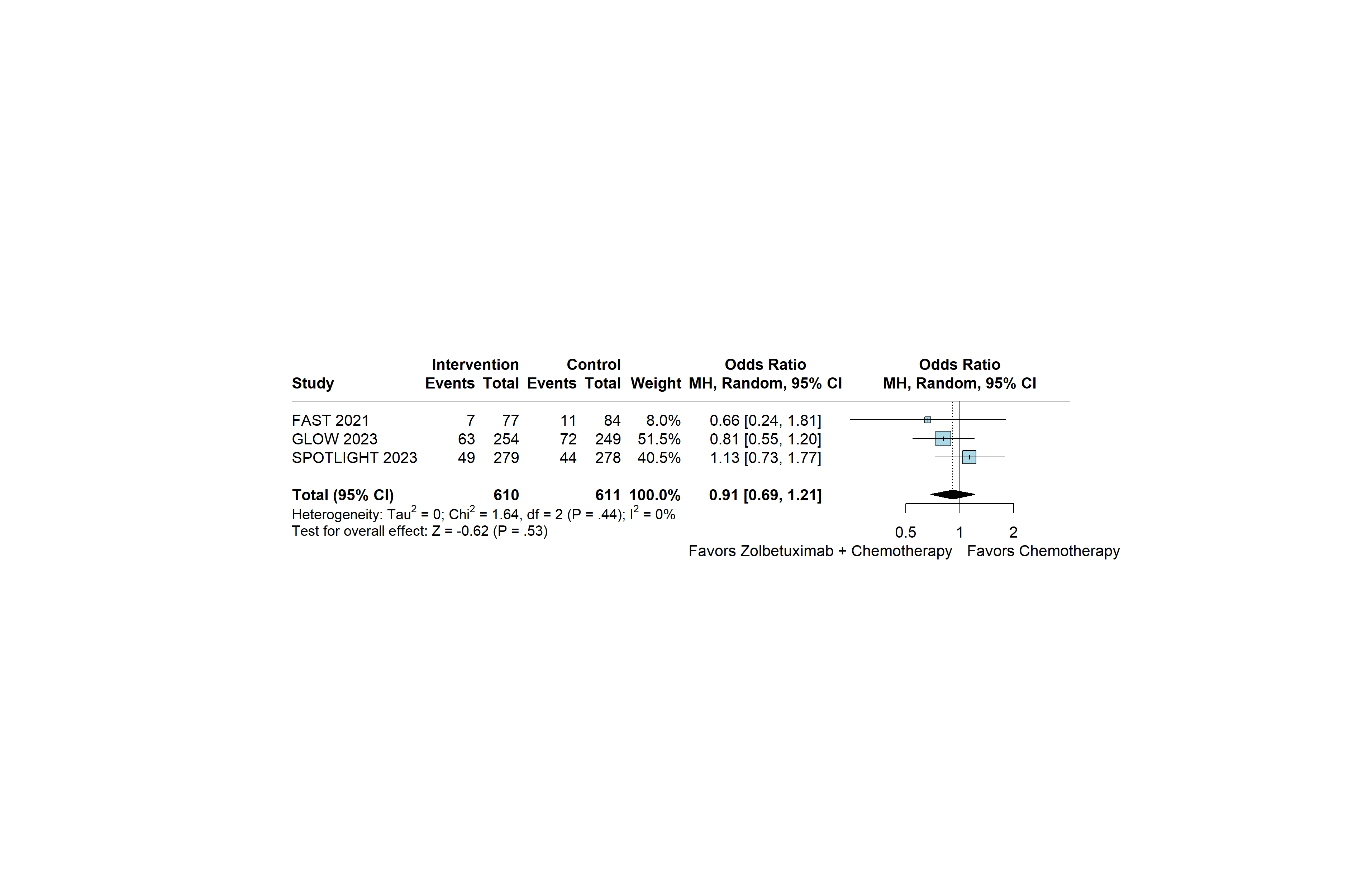


**Supplementary Figure S11.** Any grade of alanine aminotransferase increased.


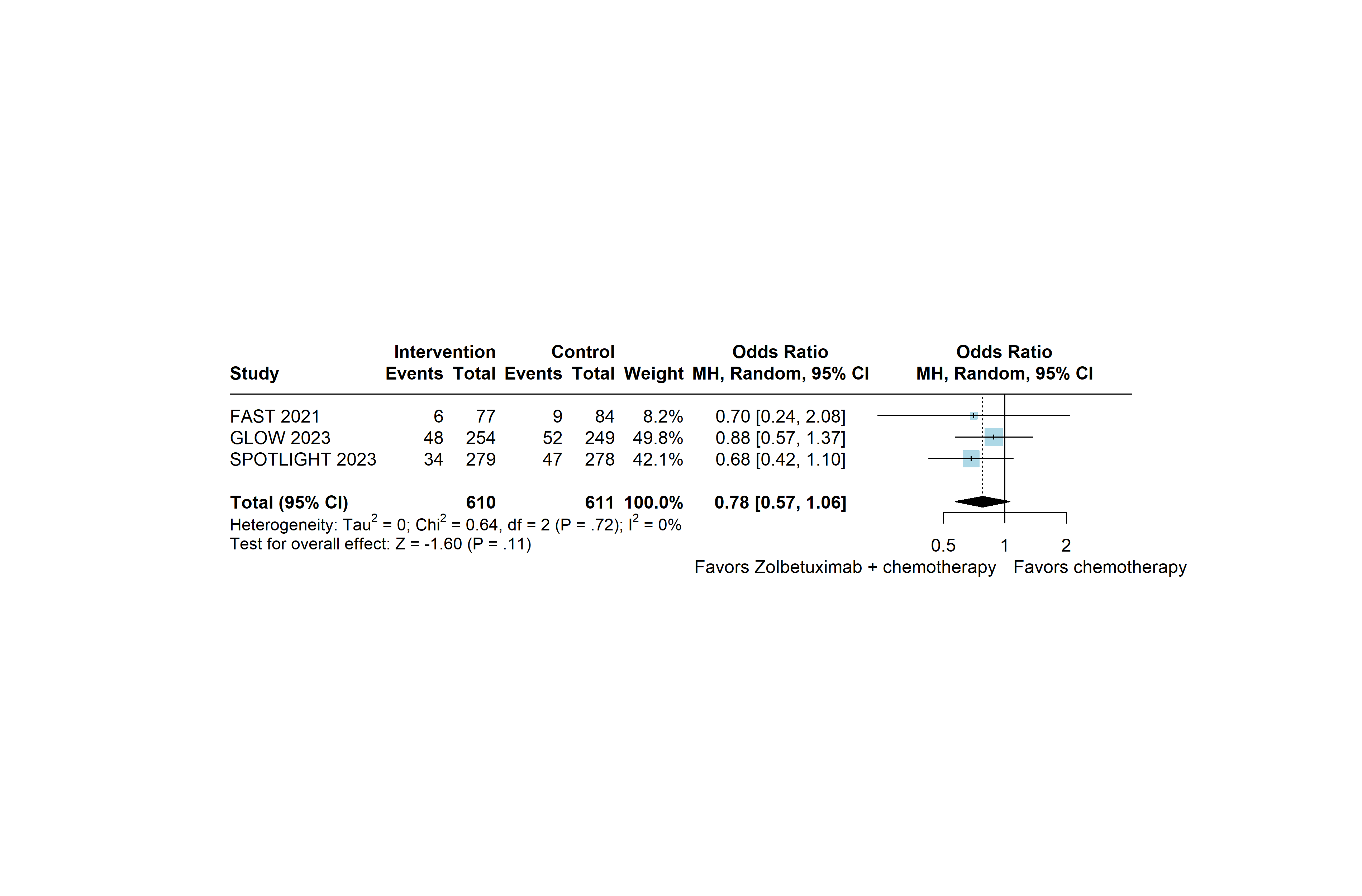


**Supplementary Figure S12.** Any grade of thrombocytopenia.


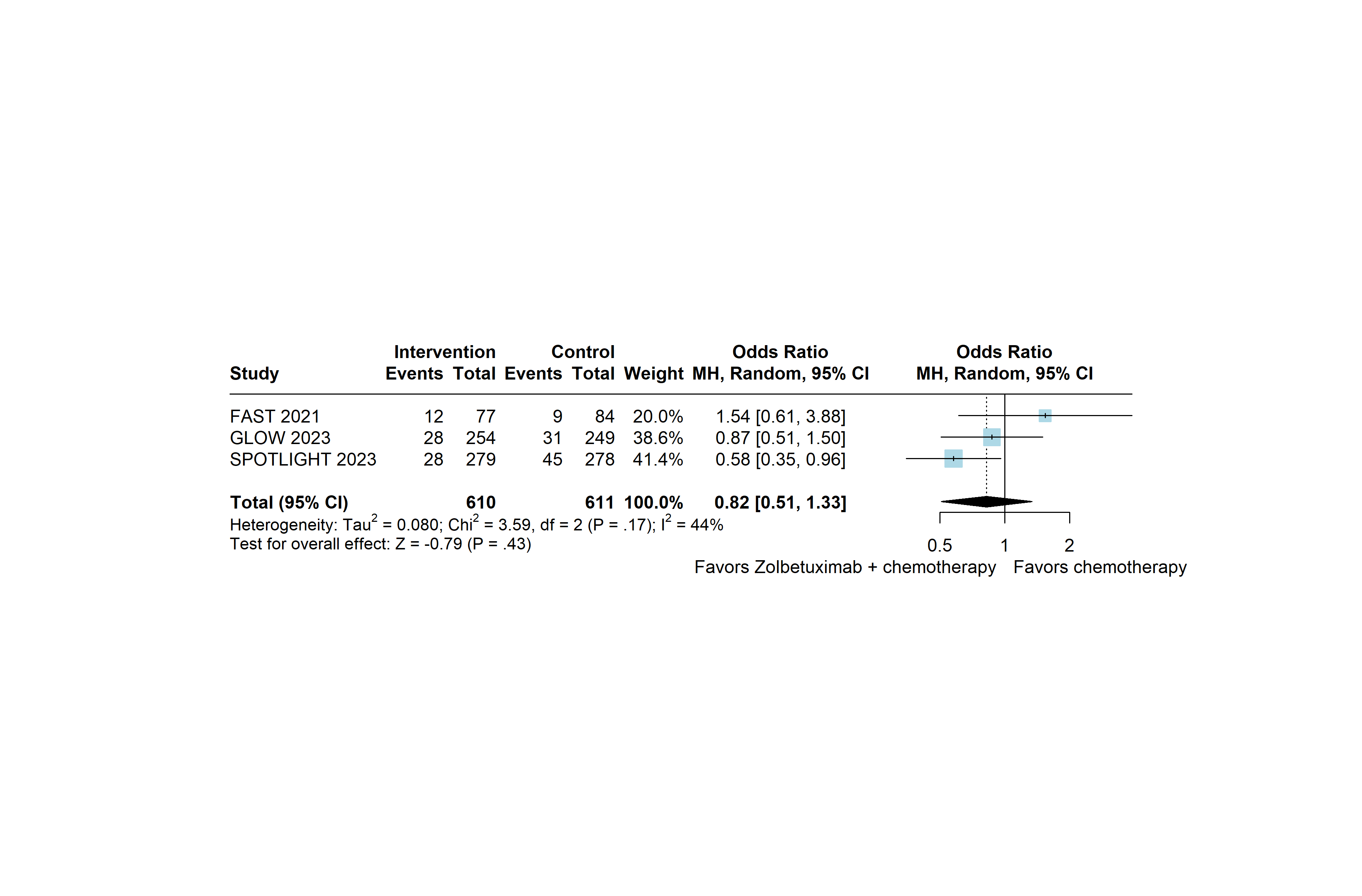


**Supplementary Figure S13.** Grade ≥3 of all treatment-emergent events.

**
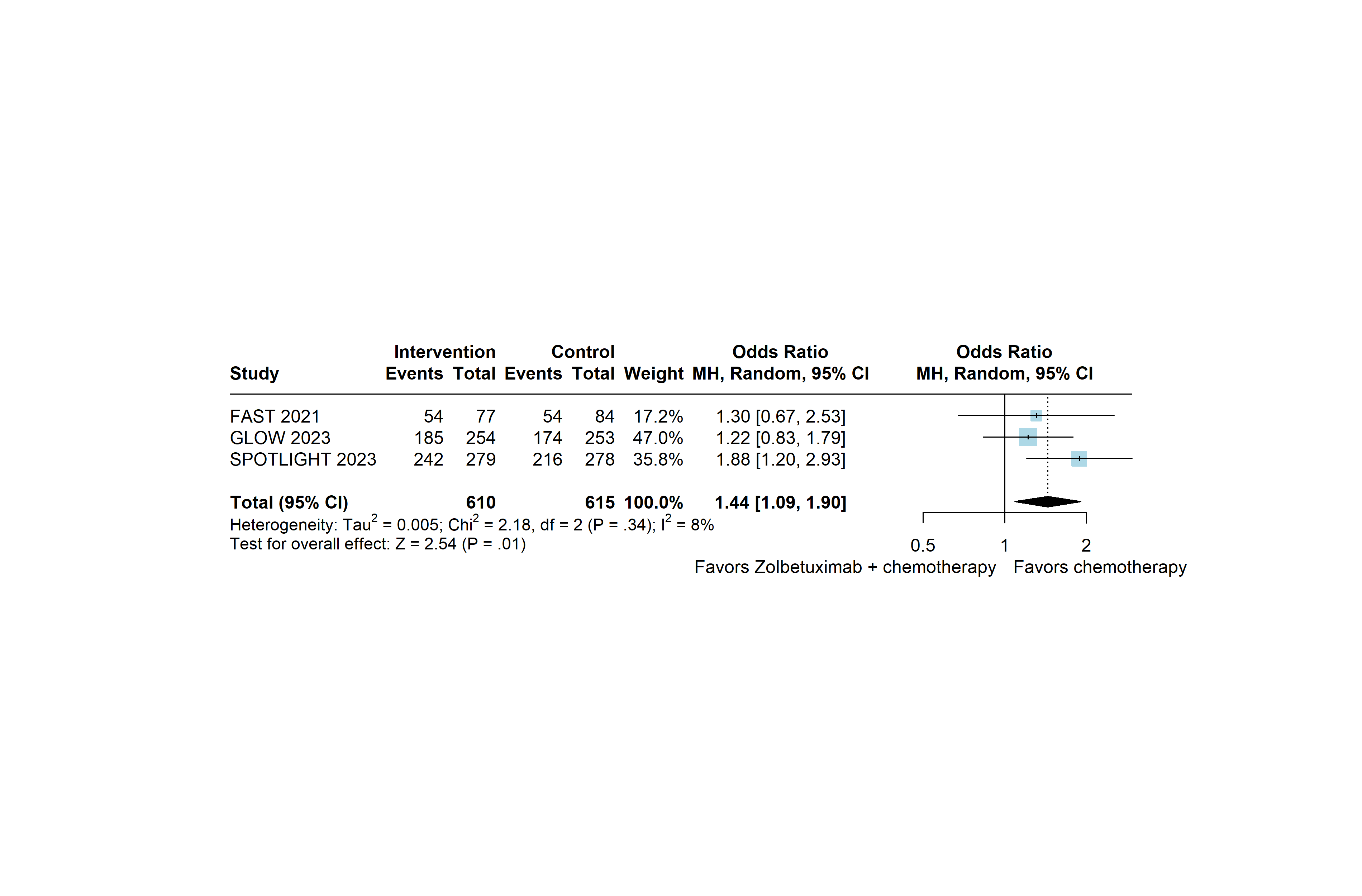
**

**Supplementary Figure S14.** Grade ≥3 of nausea.

**
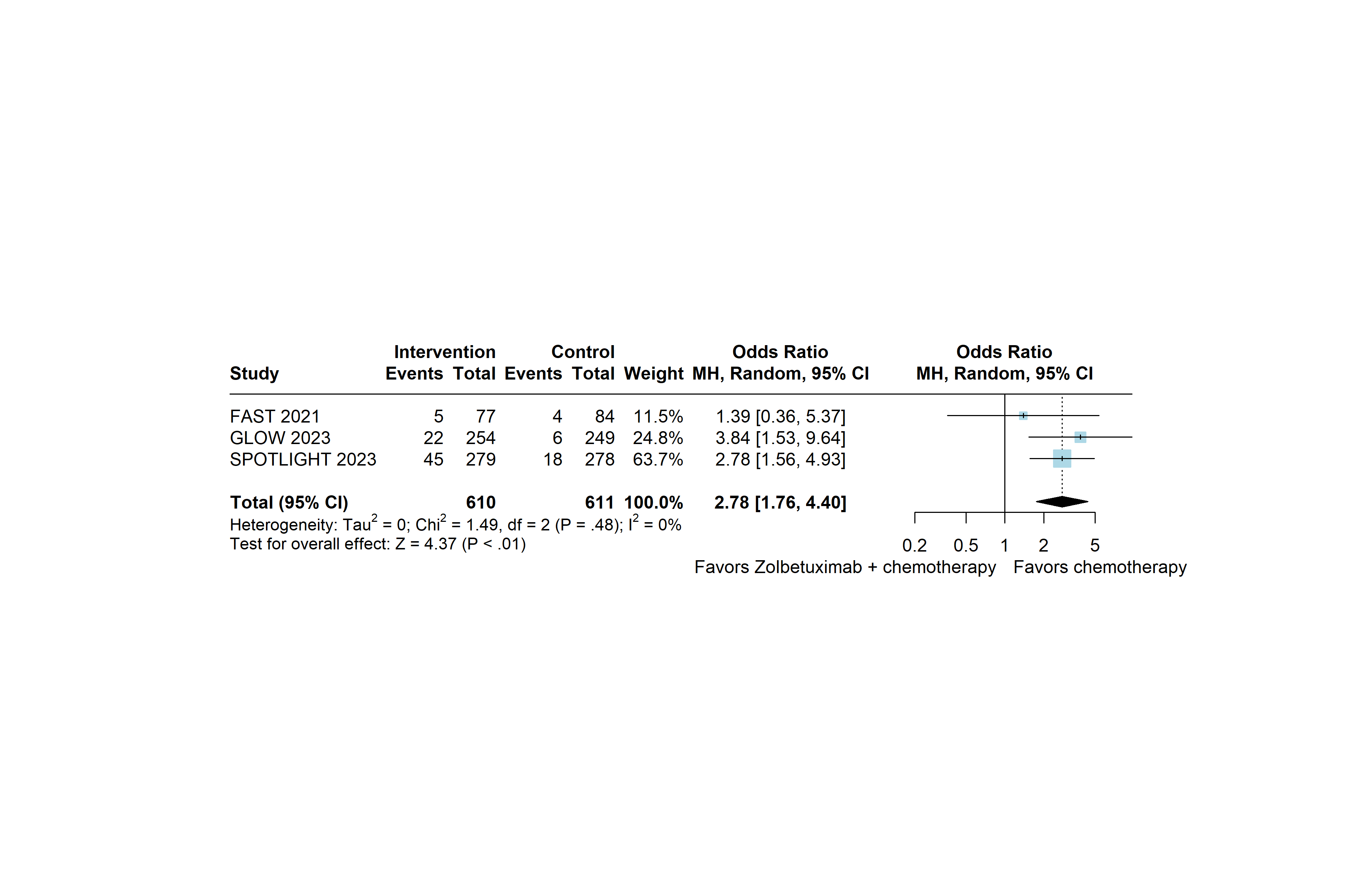
**

**Supplementary Figure S15.** Grade ≥3 of vomiting.

**
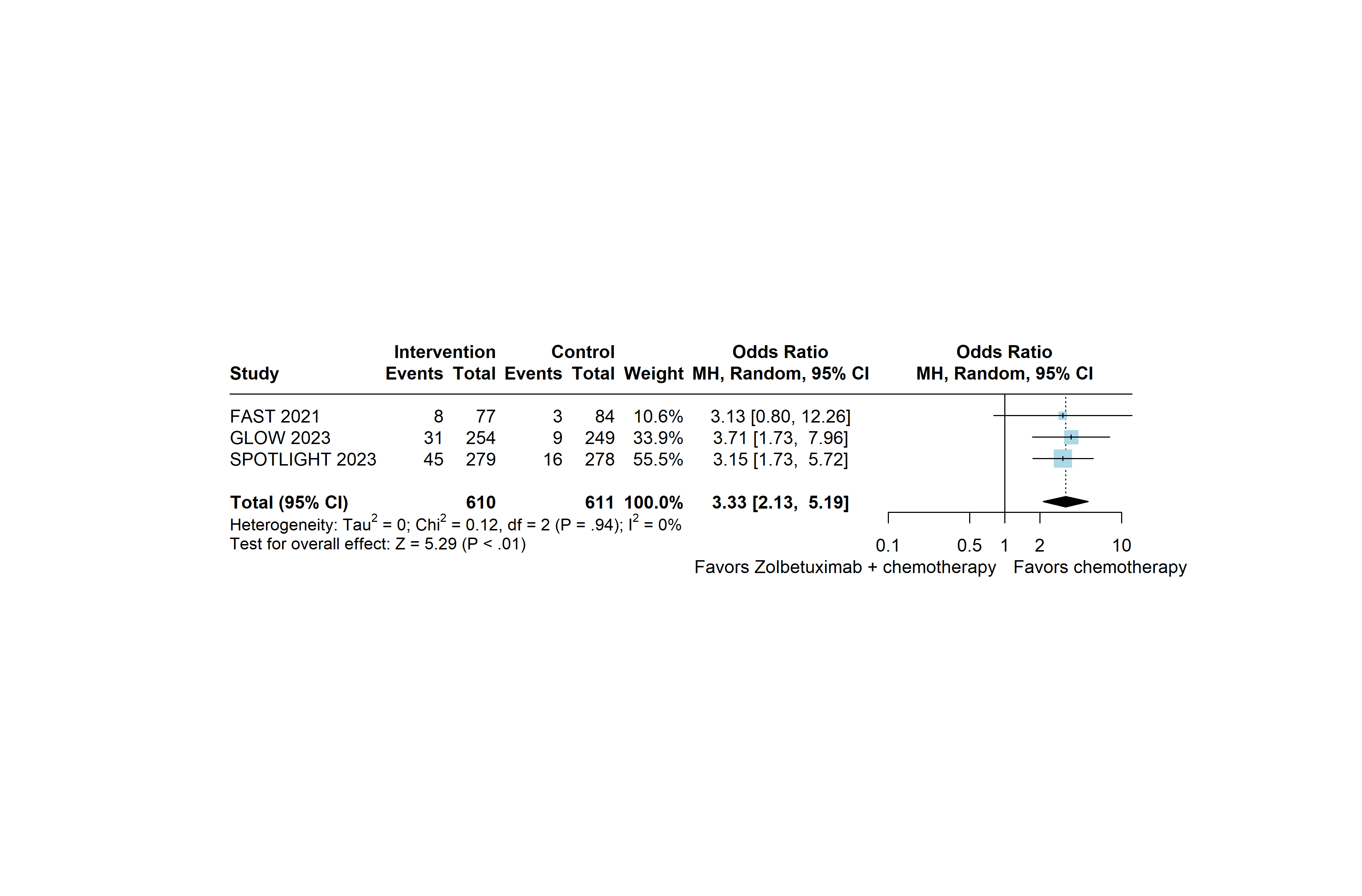
**

**Supplementary Figure S16.** Grade ≥3 of neutropenia.


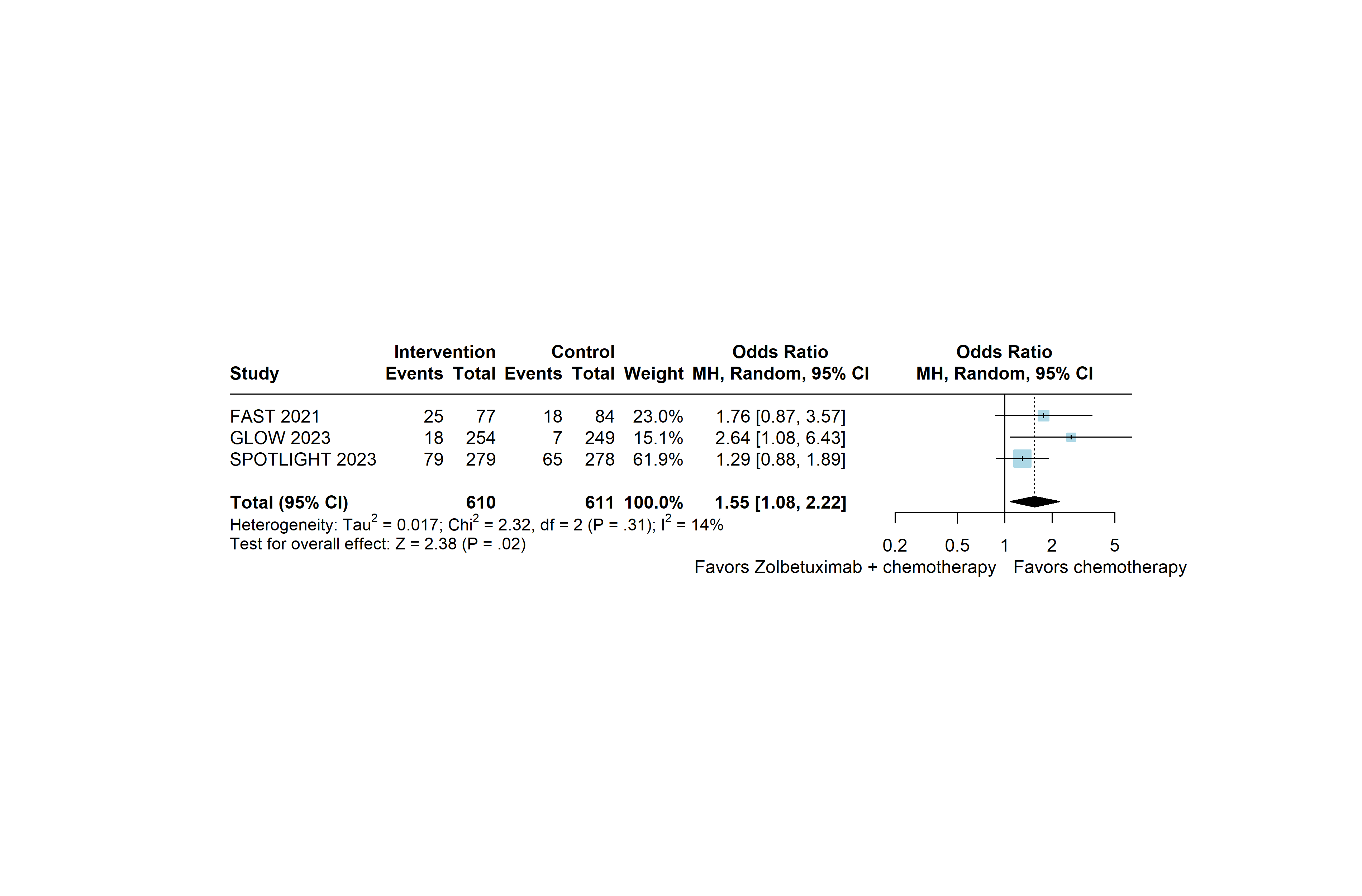


**Supplementary Figure S17.** Grade ≥3 of asthenia.


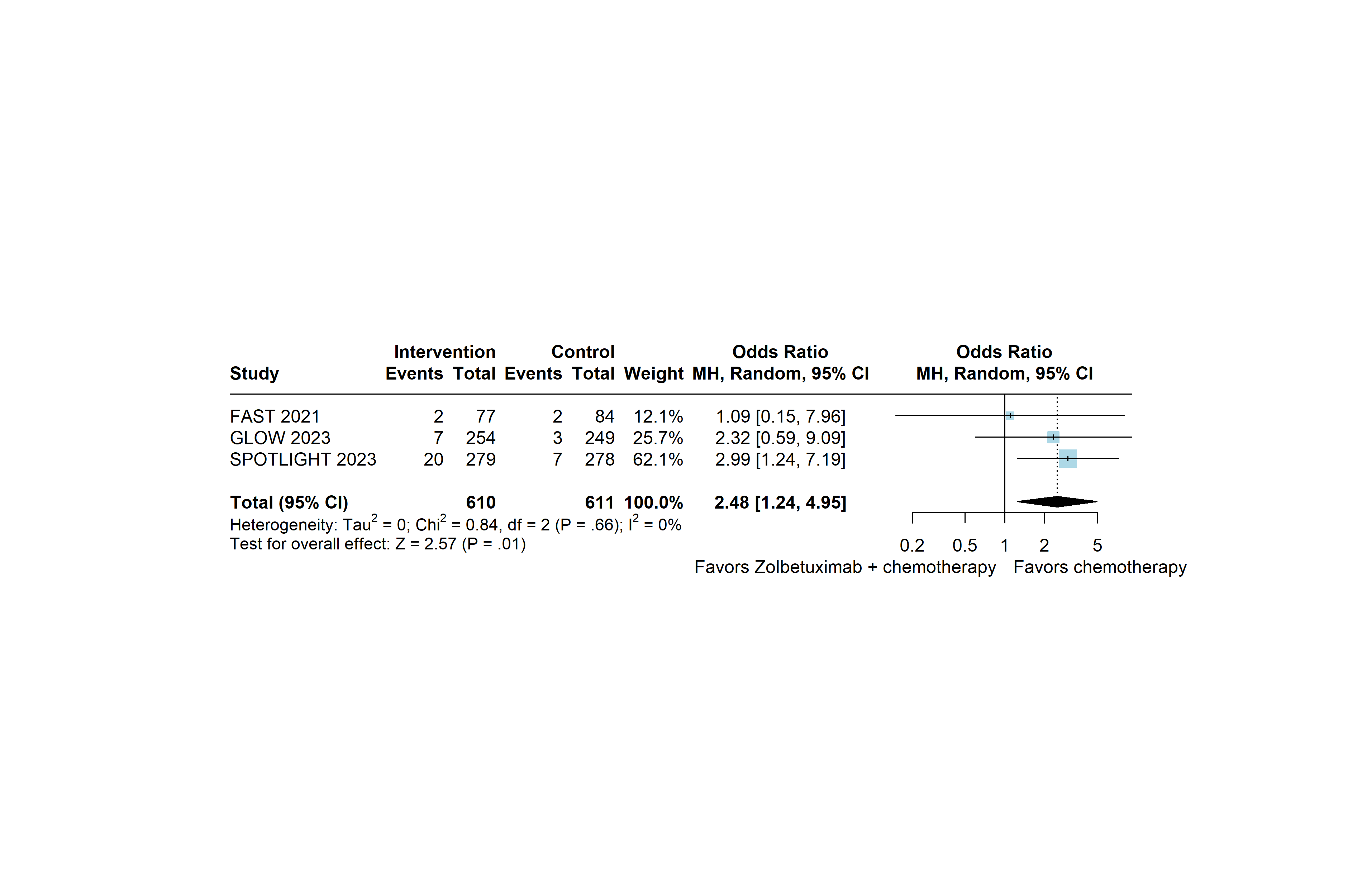


**Supplementary Figure S18.** Grade ≥3 of weight decrease.


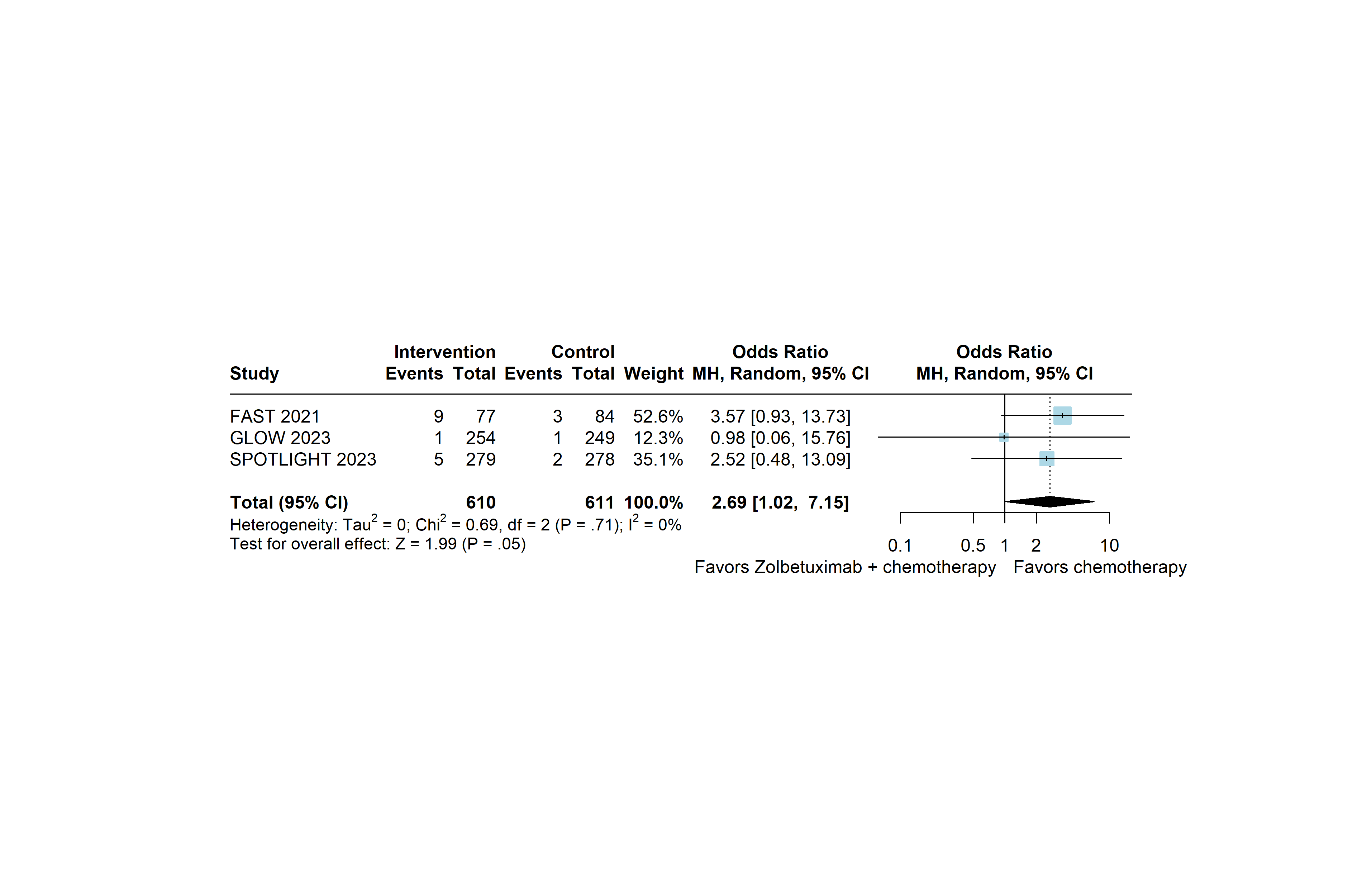


**Supplementary Figure S19.** Grade ≥3 of decrease appetite.

**
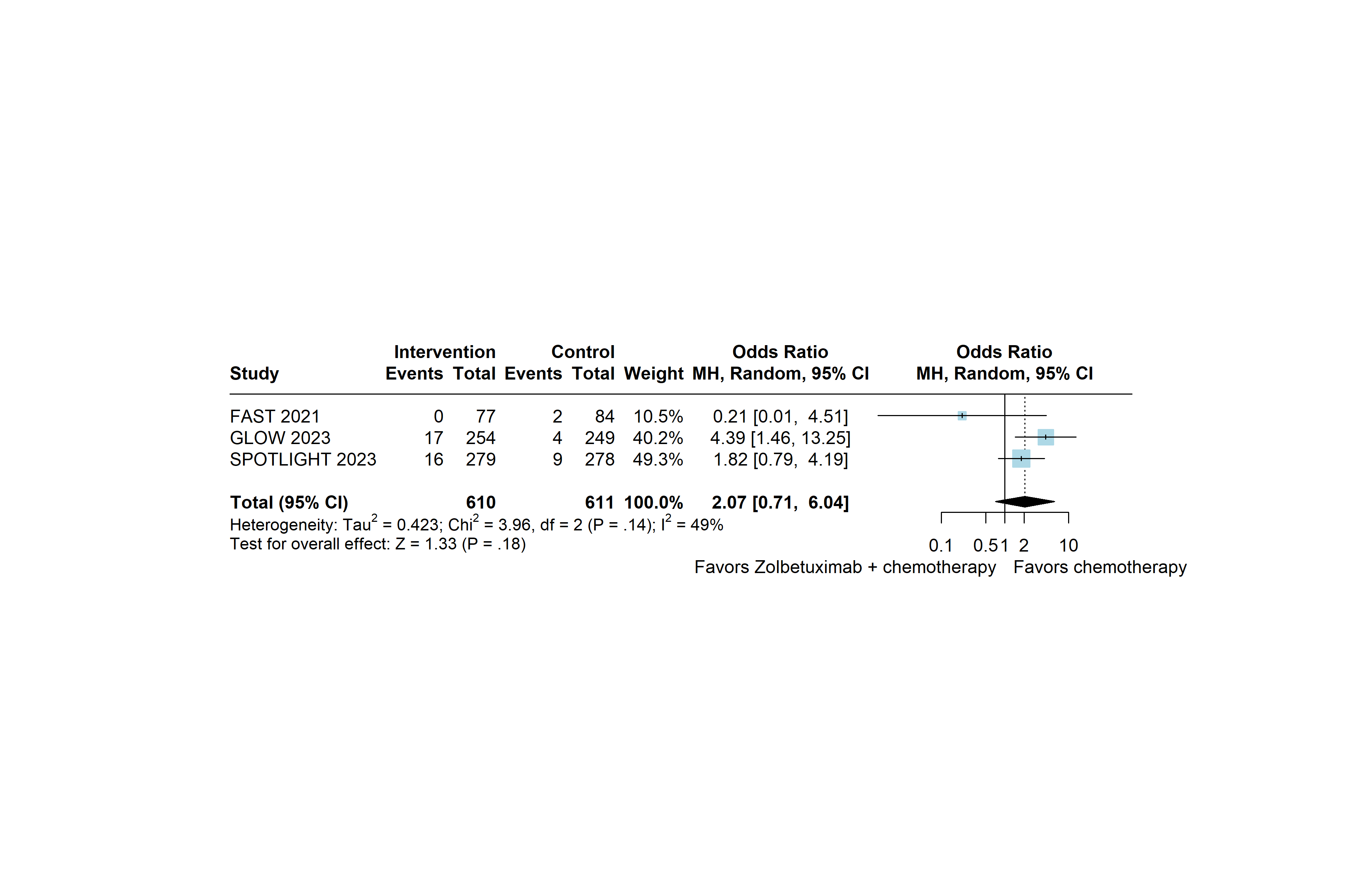
**

**Supplementary Figure S20.** Grade ≥3 of diarrhea.


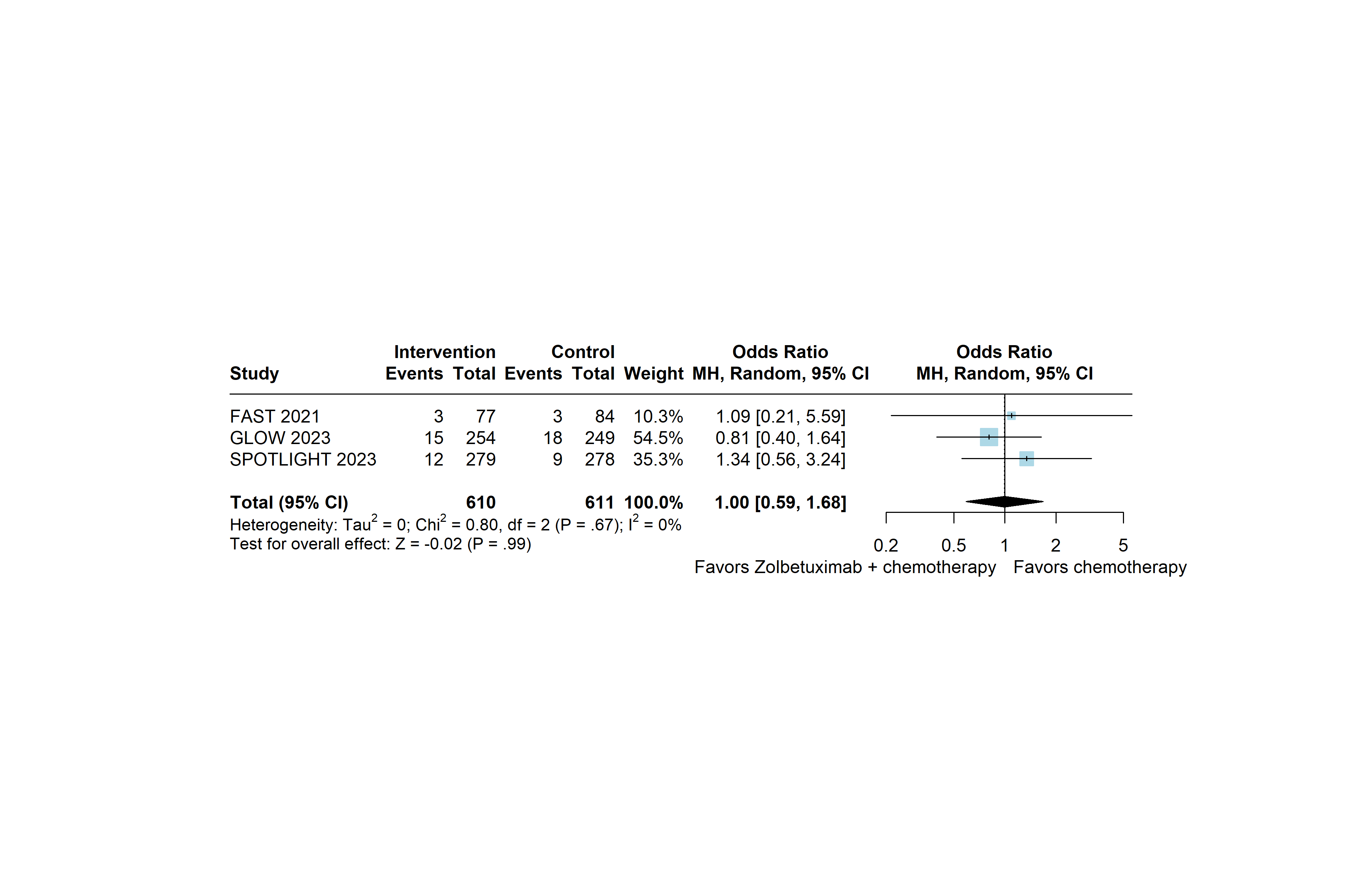


**Supplementary Figure S21.** Grade ≥3 of anemia.


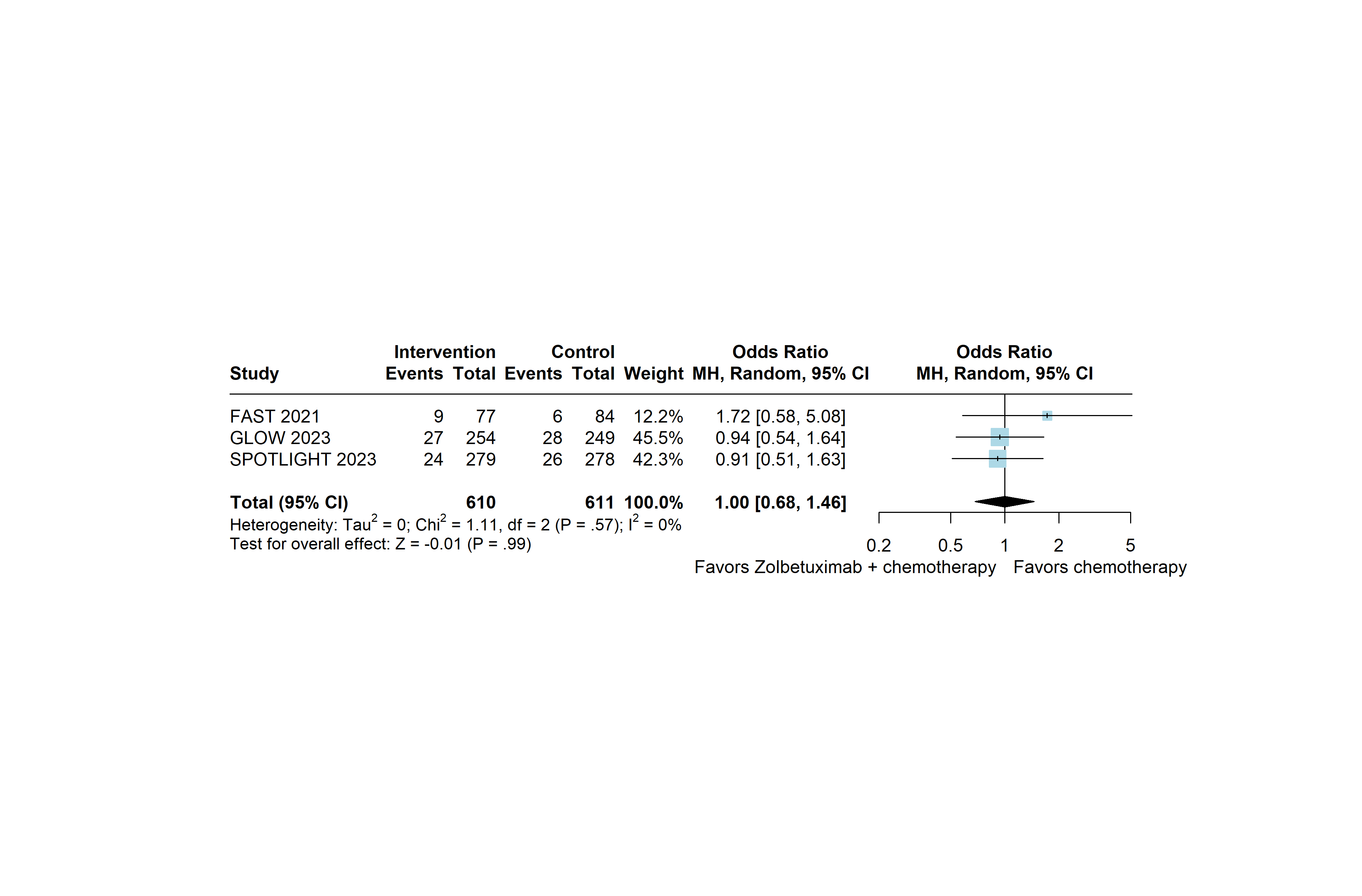


**Supplementary Figure S22.** Grade ≥3 of fatigue.


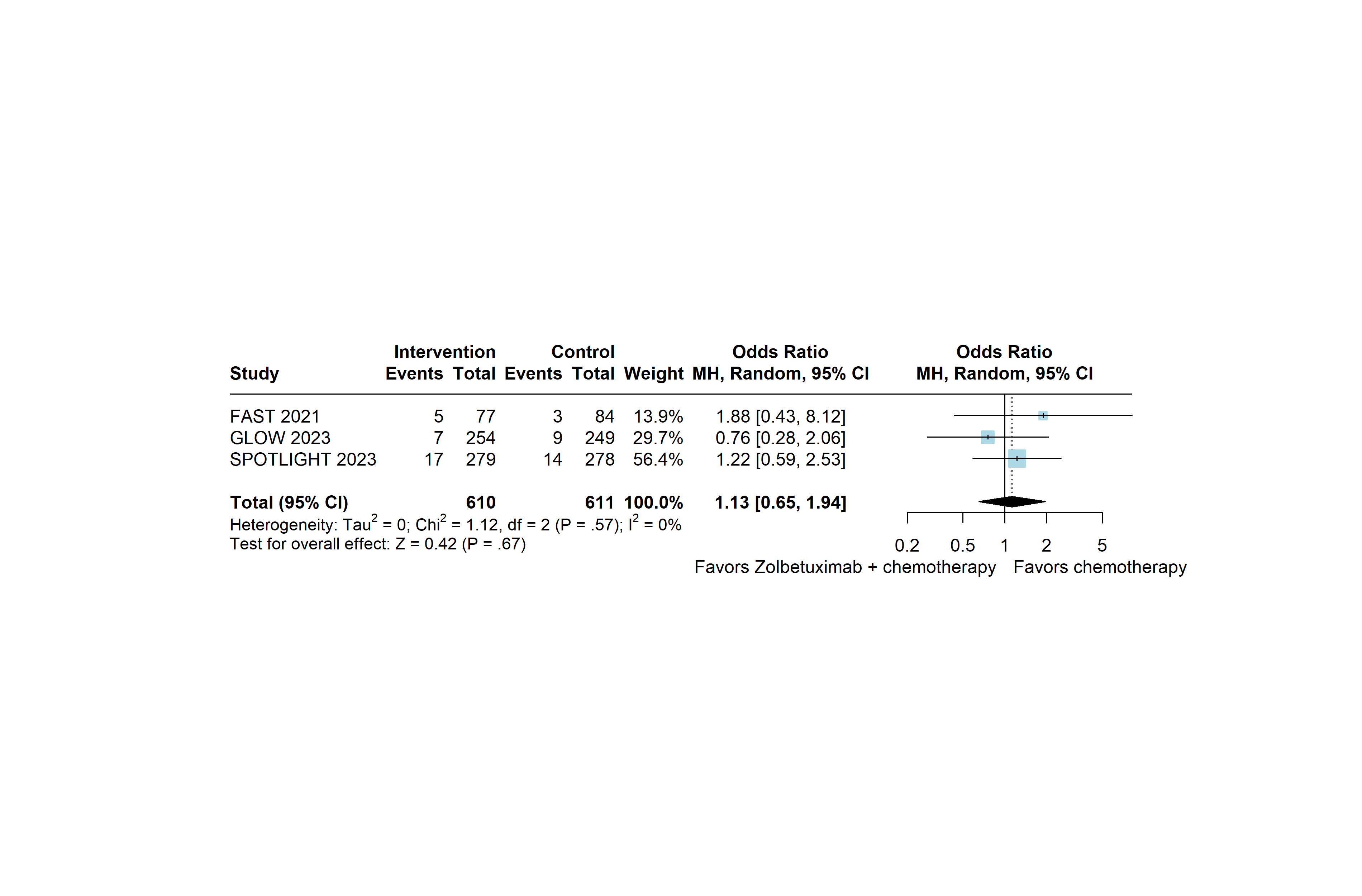


**Supplementary Figure S23.** Grade ≥3 of abdominal pain.


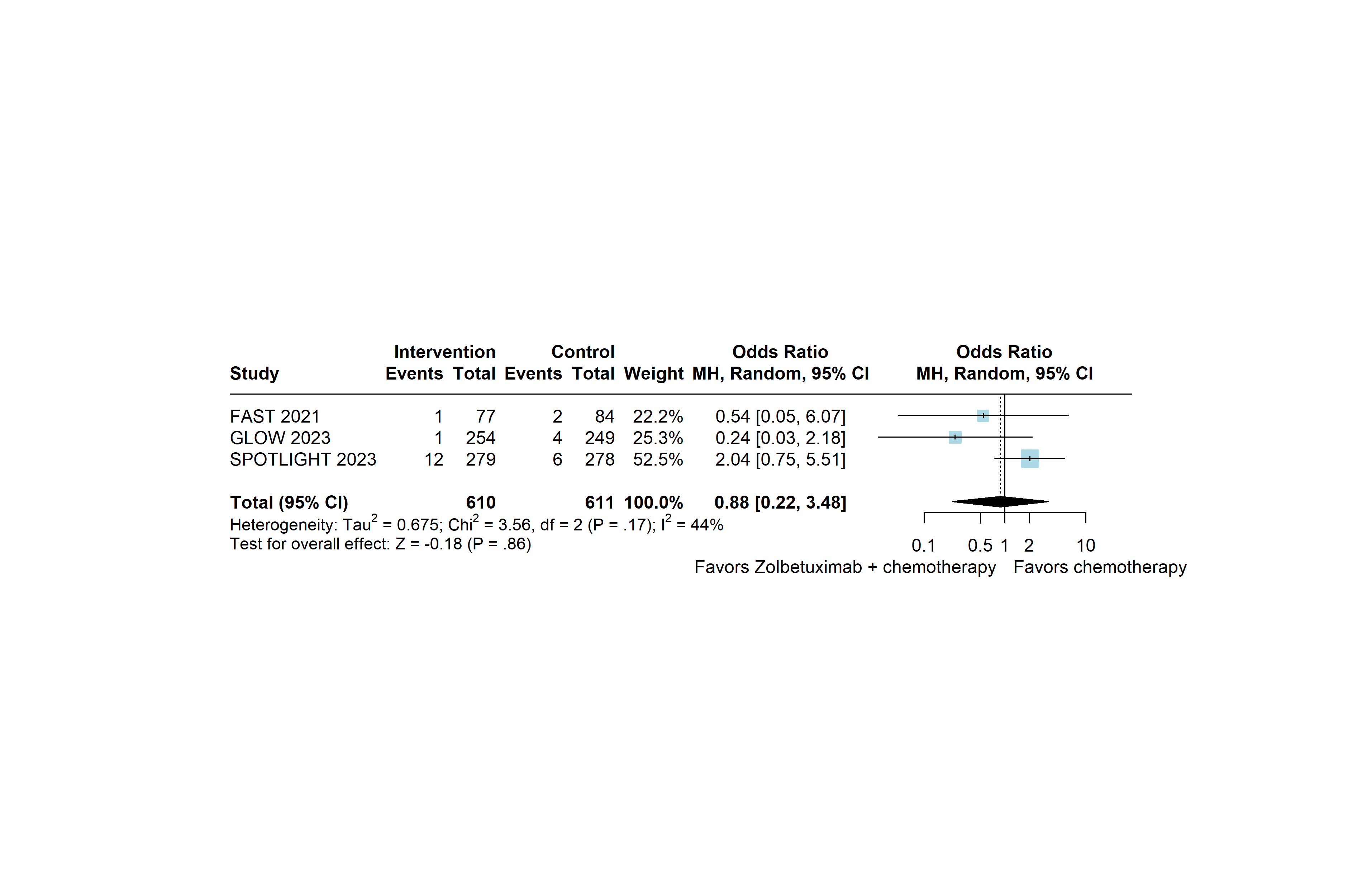


**Supplementary Figure S24.** Grade ≥3 of pyrexia.


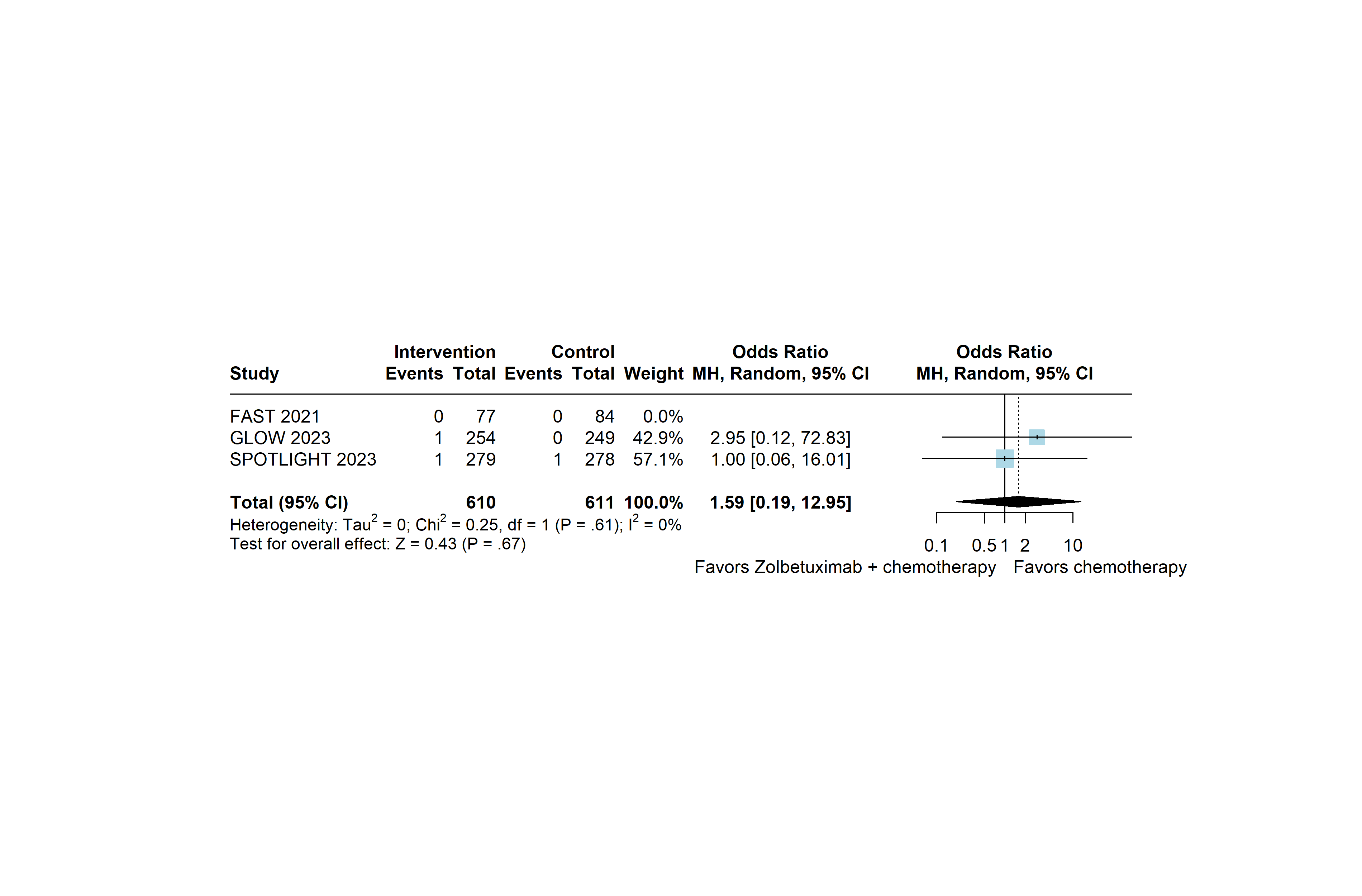


**Supplementary Figure S25.** Grade ≥3 of oedema peripheral.


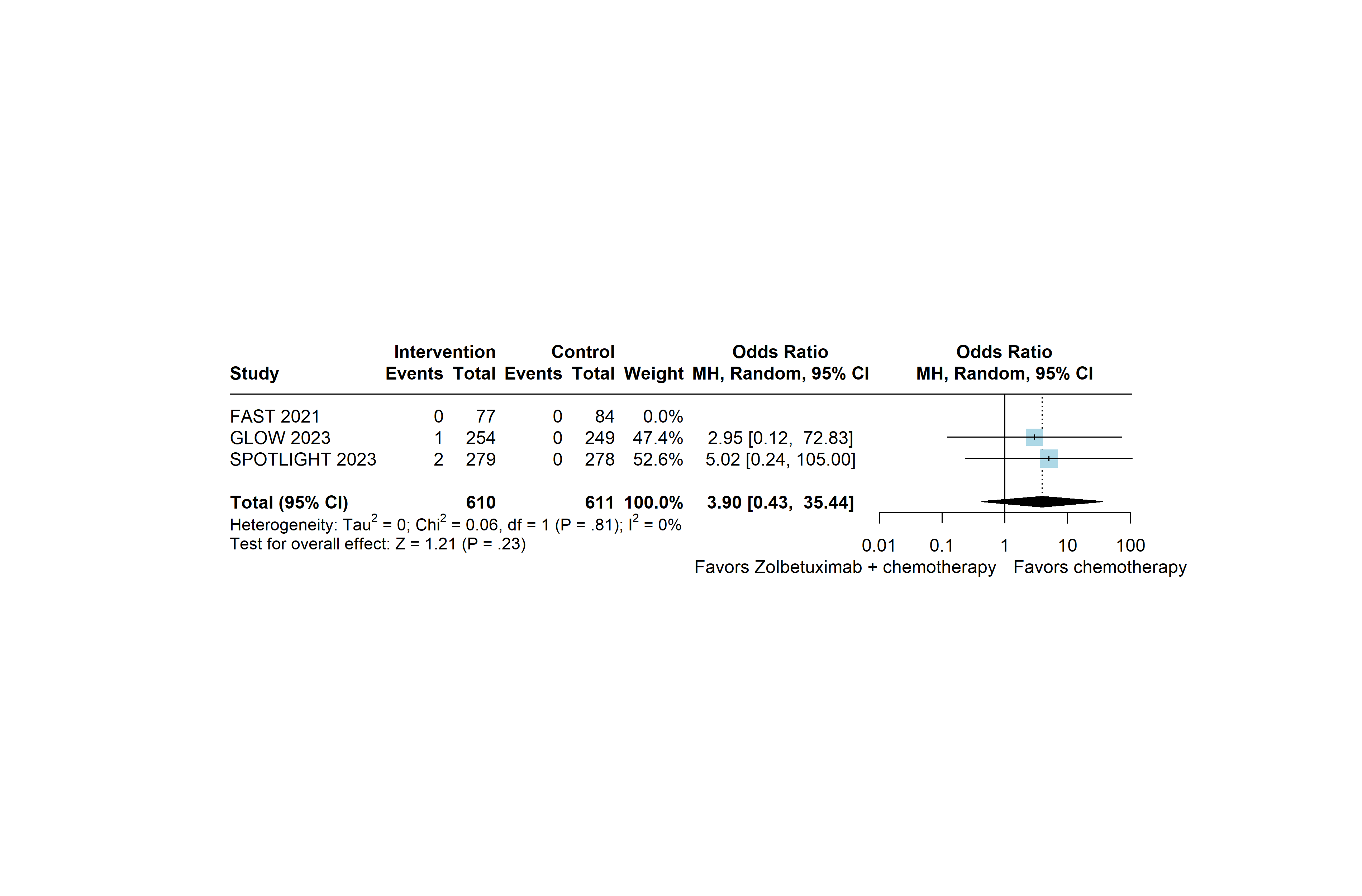


**Supplementary Figure S26.** Grade ≥3 of aspartate aminotransferase increased.


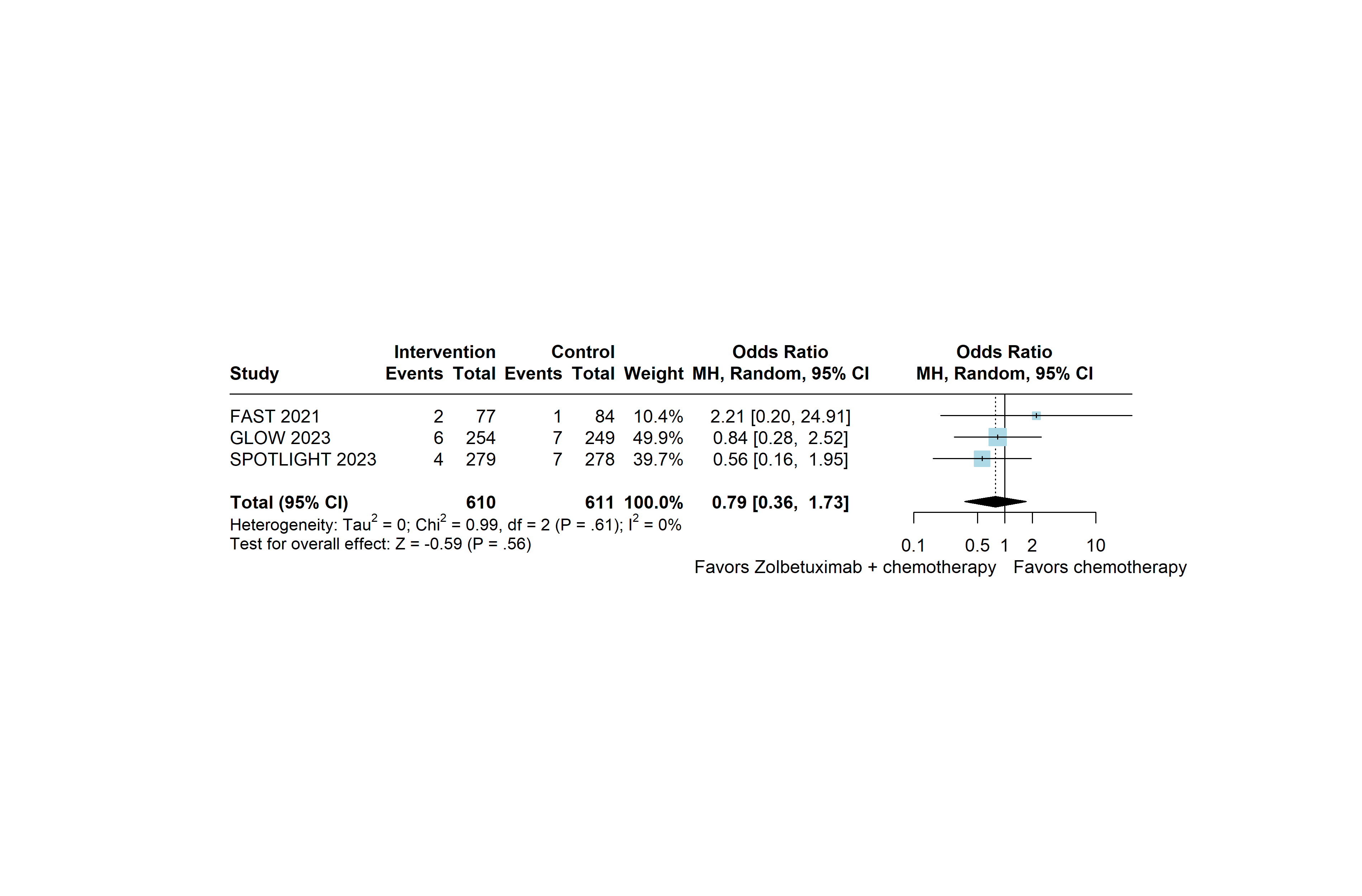


**Supplementary Figure S27.** Grade ≥3 of alanine aminotransferase increased.


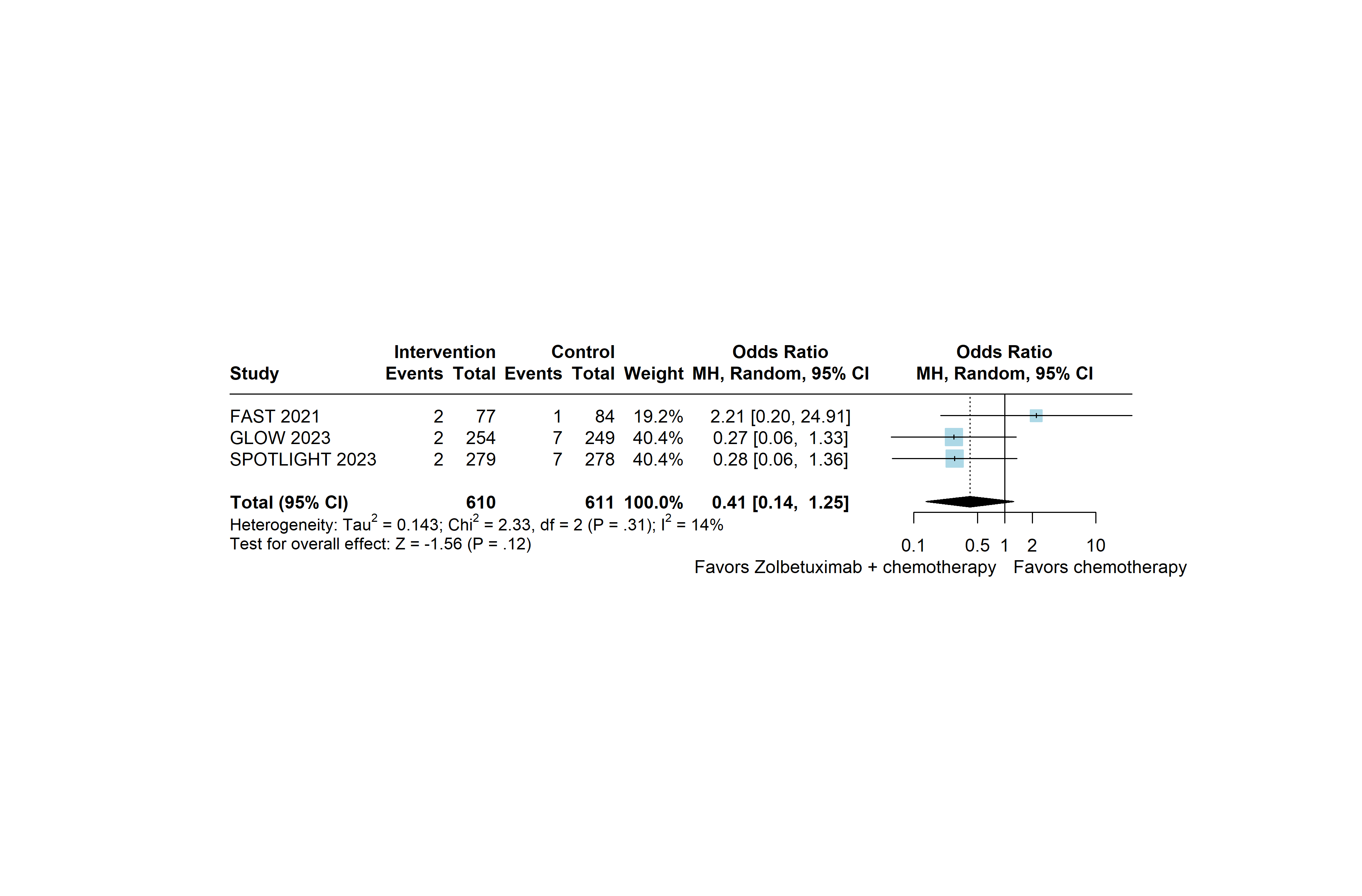


**Supplementary Figure S28.** Grade ≥3 of thrombocytopenia.


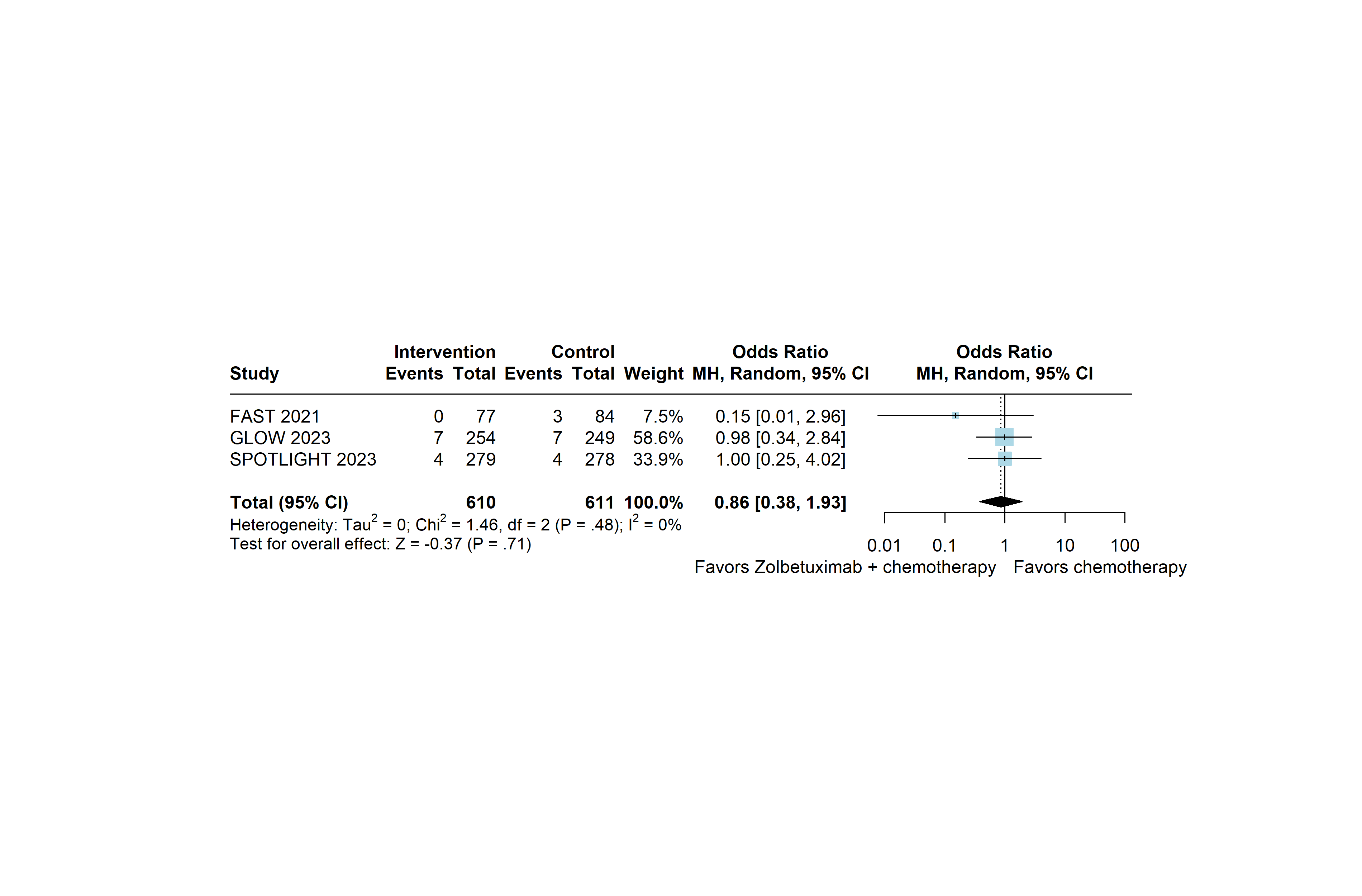


**Supplementary Figure S29.** Leave-one-out sensitivity analyses. **A.** Progression-free survival. **B.** Overall survival.


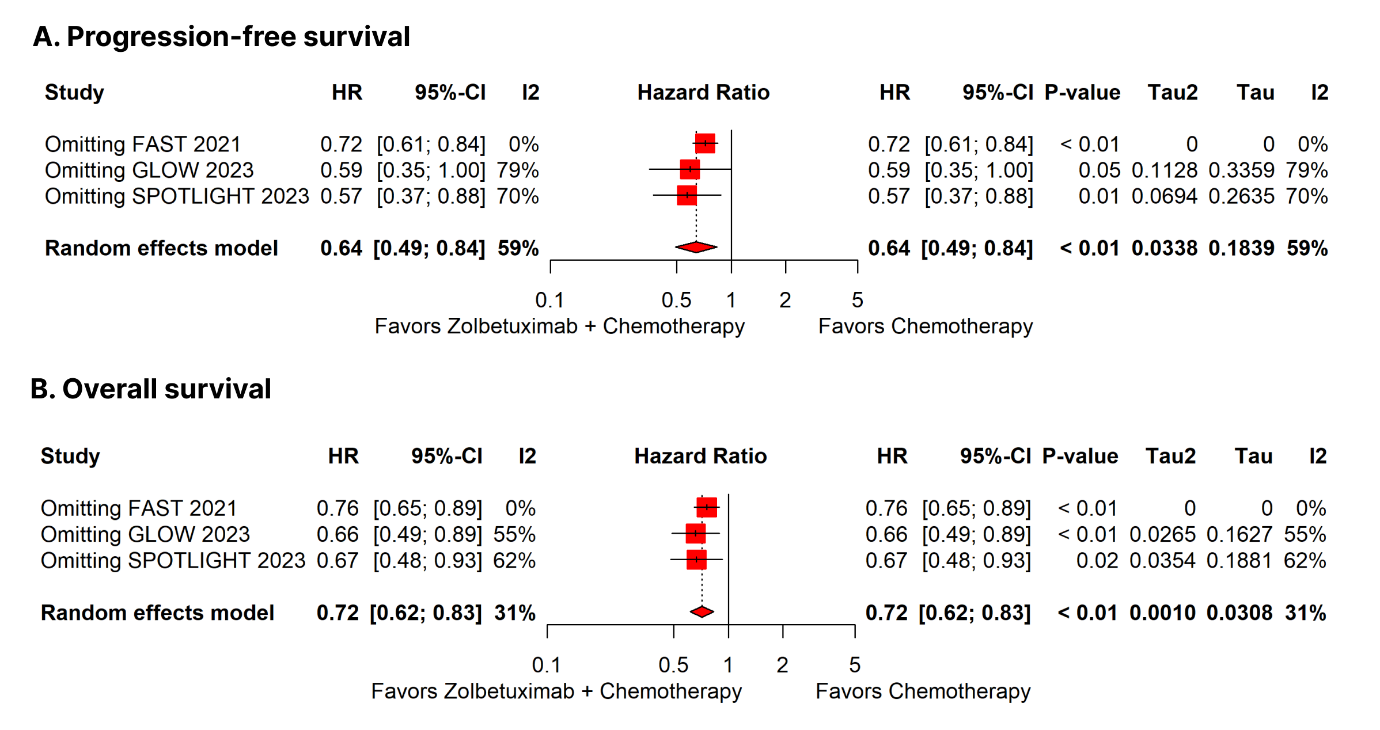


**Supplementary Figure S30.** Critical appraisal of RCTs according to the Cochrane Collaboration’s tool for assessing risk of bias in randomized trials.


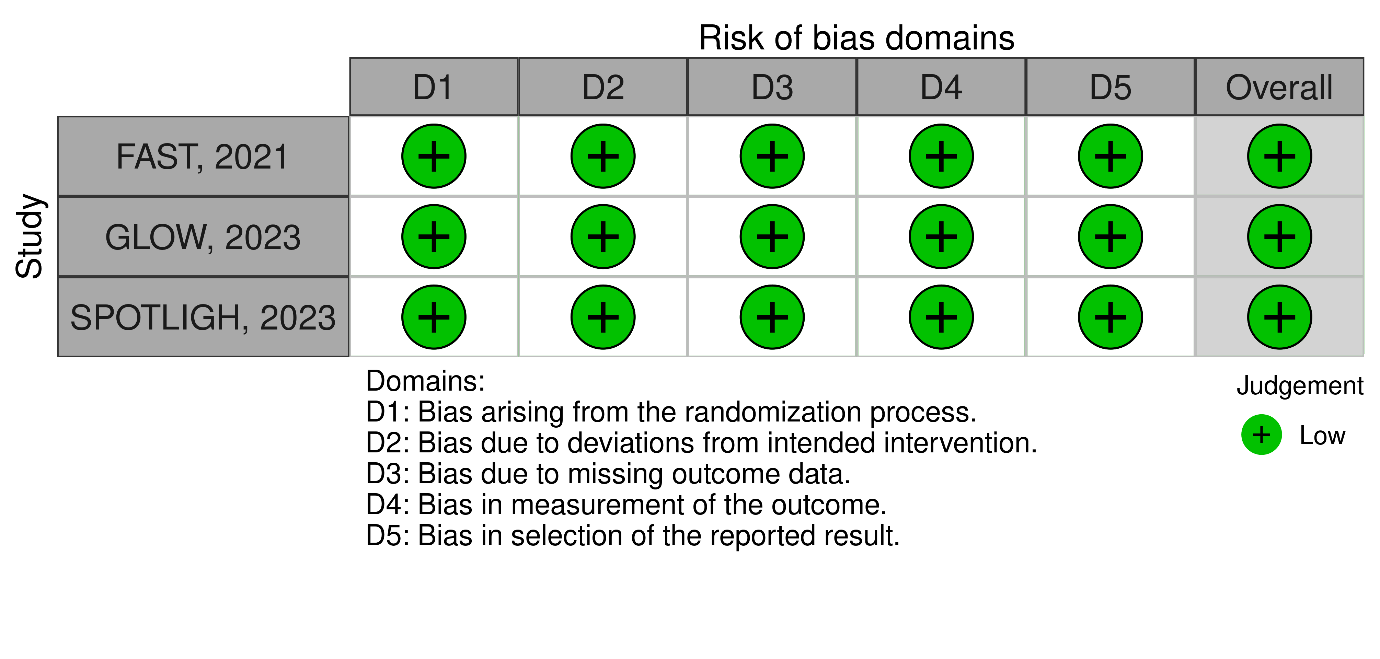

Supplement: Supplementary file 1 — Supplementary Material 1 [file 12885_2024_11980_MOESM1_ESM.docx]
